# MARCH8 Ubiquitinates the Hepatitis C Virus Nonstructural 2 Protein and Mediates Viral Envelopment

**DOI:** 10.1016/j.celrep.2019.01.075

**Published:** 2019-02-12

**Authors:** Sathish Kumar, Rina Barouch-Bentov, Fei Xiao, Stanford Schor, Szuyuan Pu, Elise Biquand, Albert Lu, Brett D. Lindenbach, Yves Jacob, Caroline Demeret, Shirit Einav

**Affiliations:** 1 Department of Medicine, Division of Infectious Diseases and Geographic Medicine, and Department of Microbiology and Immunology, Stanford University School of Medicine, Stanford, CA, USA; 2 Département de Virologie, Unité de Génétique Moléculaire des Virus ARN (GMVR), Institut Pasteur, Centre National de la Recherche Scientifique; 3 Université Paris Diderot, Paris, France; 4 Department of Biochemistry, Stanford University School of Medicine, Stanford, CA, USA; 5 Department of Microbial Pathogenesis, Yale School of Medicine, New Haven, CT, USA; 6 These authors contributed equally; 7 Lead Contact

## Abstract

The mechanisms that regulate envelopment of HCV and other viruses that bud intracellularly and/or lack late-domain motifs are largely unknown. We reported that K63 polyubiquitination of the HCV nonstructural (NS) 2 protein mediates HRS (ESCRT-0 component) binding and envelopment. Nevertheless, the ubiquitin signaling that governs NS2 ubiquitination remained unknown. Here, we map the NS2 interactome with the ubiquitin proteasome system (UPS) via mammalian cell-based screens. NS2 interacts with E3 ligases, deubiquitinases, and ligase regulators, some of which are candidate proviral or antiviral factors. MARCH8, a RING-finger E3 ligase, catalyzes K63-linked NS2 polyubiquitination *in vitro* and in HCV-infected cells. MARCH8 is required for infection with HCV, dengue, and Zika viruses and specifically mediates HCV envelopment. Our data reveal regulation of HCV envelopment via ubiquitin signaling and both a viral protein substrate and a ubiquitin K63-linkage of the understudied MARCH8, with potential implications for cell biology, virology, and host-targeted antiviral design.

## In Brief

The mechanisms that regulate intracellular viral envelopment are unknown. Kumar et al. report that MARCH8 catalyzes K63-linked polyubiquitination of the HCV nonstructural 2 protein and subsequently ESCRT recruitment and HCV envelopment. MARCH8 is required for infection with other Flaviviridae family members, thereby representing a potential host target for antiviral strategies.

## Graphical Abstract

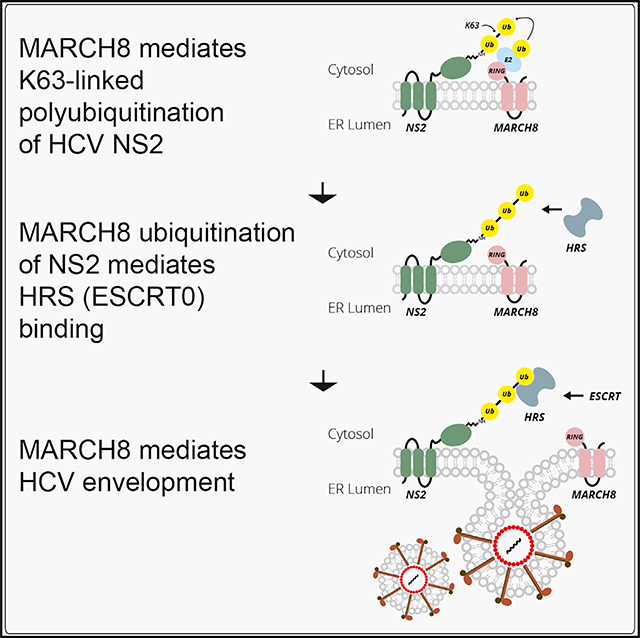

## INTRODUCTION

Viruses commonly acquire their envelopes at the plasma membrane by recruiting the host endosomal sorting complex required for transport (ESCRT) machinery via conserved motifs, designated late domains (Votteler and Sundquist, 2013). However, the mechanism underlying intracellular envelopment of some RNA viruses, such as *Flaviviridae*, and envelopment of viruses lacking defined late-domain motifs are poorly characterized.

The *Flaviviridae* is a family of enveloped, positive-strand RNA viruses that include the *Hepacivirus* hepatitis C virus (HCV), a major cause of liver disease, and the flaviviruses dengue (DENV) and Zika (ZIKV), two global health threats. Although no antiviral drugs are approved for treatment of flavivirus infections, effective, direct-acting antivirals are approved for HCV treatment. Nevertheless, limited access to those drugs and viral resistance continue to challenge current efforts to eradicate HCV (Zoulim et al., 2015).

The HCV core protein and E1 and E2 glycoproteins form new virions, whereas the nonstructural (NS) proteins NS3, 4A, 4B, 5A, and 5B form the viral replication machinery, and p7 and NS2 are essential for infectious virus production (Gentzsch et al., 2013; Jirasko et al., 2010; Jones et al., 2007; Steinmann et al., 2007). The model of HCV production suggests that assembly of viral particles begins on or near the surface of lipid droplets (Bartenschlager et al., 2011), followed by budding into the endoplasmic reticulum (ER; where the envelope glycoproteins are retained) and release of enveloped, infectious virions via the secretory pathway (Coller et al., 2012; Jones and McLauchlan, 2010; Roingeard et al., 2008). This process requires coordination of all 10 HCV proteins, along with multiple host factors (Bartenschlager et al., 2011). NS2, in particular, has a critical role in early viral assembly, envelopment, maturation, and release (de la Fuente et al., 2013; Dentzer et al., 2009; Jirasko et al., 2010; Jones et al., 2007; Popescu et al., 2011). However, a comprehensive understanding of the mechanisms that govern the roles of NS2 in HCV assembly, and especially in envelopment, is still lacking.

Ubiquitination is a post-translational modification that controls various cellular processes, such as protein degradation, signal transduction, translocation across membranes, and intracellular membrane trafficking (Chen and Sun, 2009). The sequential process of ubiquitination starts with activation of ubiquitin by an E1 activating enzyme, followed by transfer of the activated ubiquitin to an E2 ubiquitin-conjugating enzyme and ubiquitin transfer to a substrate by an E3 ligase. E3 ligases are categorized based on the mechanism of ubiquitin transfer into RING (really interesting new gene), HECT (homologous to the E6AP carboxyl terminus), and RBR (RING-between RING-RING) families (Metzger et al., 2014). RING E3 ligases contain a RING finger domain, which brings together the substrate and the E2-ubiquitin and catalyzes the ligation (Metzger et al., 2014). They act either as multi-protein complexes, exemplified by the cullin-based RING-E3 ligases (CRLs), or as monomers or dimers (RING-E3). Among the latter group, the MARCH (membrane-associated RING-CH) family consists of 11 mammalian E3 ligases that harbor a catalytic domain with a C4HC3 cysteine-histidine (RINGCH finger) configuration in their N-terminal cytoplasmic tail and transmembrane domains (Samji et al., 2014). MARCH proteins reside in various intracellular compartments and affect the trafficking of membrane molecules (Samji et al., 2014). Specifically, MARCH8 is located on endosomes and the plasma membrane and regulates the subcellular localization of its substrates (Eyster et al., 2011; Roy et al., 2017; Samji et al., 2014). The function of endogenous MARCH8 remains largely unknown, and overall, this E3 ligase family is understudied.

Enveloped RNA viruses commonly recruit TSG101, Nedd4-like E3 ubiquitin ligases, or Alix to enter the ESCRT network via late domains and bud from the plasma membrane (Votteler and Sundquist, 2013). In contrast, we reported that HRS (hepatocyte growth factor-regulated tyrosine kinase substrate) serves as the entry point for HCV, a virus lacking defined late domains, into the ESCRT pathway and has a role in HCV envelopment (Barouch-Bentov et al., 2016). Moreover, we demonstrated that K63 polyubiquitination of lysine residues within HCV NS2 mediates HRS binding and HCV assembly, thereby compensating for the absence of late domains (Barouch-Bentov et al., 2016). Nevertheless, the interaction landscape of ubiquitin signaling that regulates NS2 ubiquitination and HCV envelopment and the precise E3 ubiquitin ligase remained unknown.

To address this gap in knowledge, we screened for HCV NS2 interactions with ~550 ubiquitin signaling proteins by mammalian cell-based, high-throughput, *Gaussia princeps* split-luciferase complementation assay (HT-GPCA; Biquand et al., 2017). Thirty interactions with E3 ligases were identified and validated in a secondary screen. NS2 was also found to bind three E3 ligase regulators, 4 DUBs (deubiquitinases), and an E2 ubiquitin-conjugating enzyme. Small interfering RNA (siRNA)-mediated depletion of 20 RING-E3 and HECT-E3 ligase genes identified MARCH8 as a critical factor for HCV infection. We show that MARCH8 catalyzes K63-ubiquitination of NS2 lysine residues and subsequently HCV envelopment. Lastly, we demonstrate that MARCH8 also has a role in DENV and ZIKV infections, thereby proposing a candidate, potentially “druggable” target for host-targeted antiviral strategies.

## RESULTS

### Profiling Ubiquitin Signaling Interactors of NS2 via a Proteomic Screen

**Figure 1.**
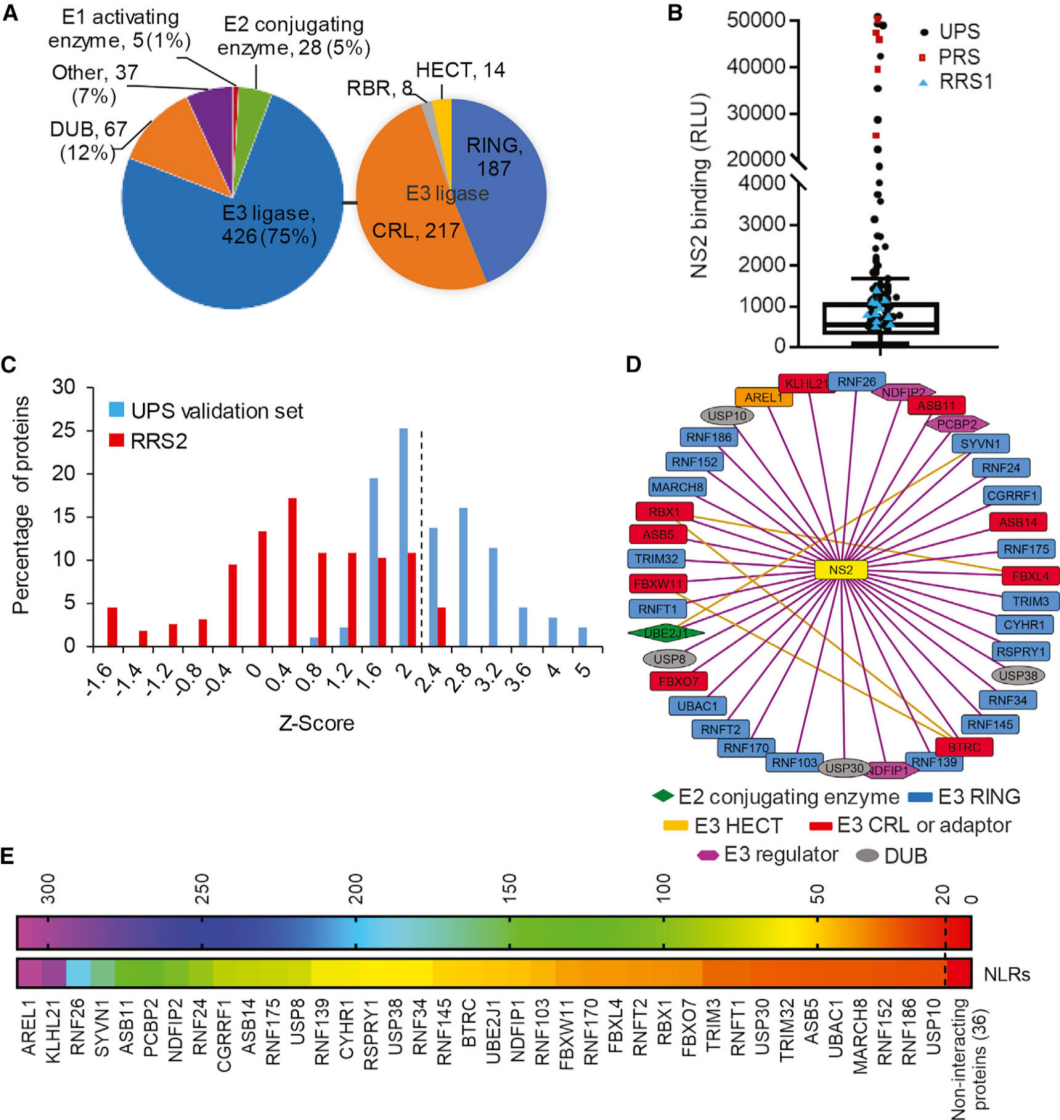
Profiling of Ubiquitin-Signaling Interactors of NS2 by HT-GPCAs (A) Pie-chart representations of the UPS library (left) and E3 ligase categories (right). The number and percentage of factors in each category are indicated. (B) Boxplot of the NS2-UPS luminescence values generated in a representative experiment out of 10 conducted in the primary screen. The box horizontal lines indicate the first, second (median), and third quartiles. Outliers above the upper whisker (Q3 + 1.5 × interquartile range [IQR]) were defined as positive interactions. Circles, squares, and triangles denote UPS, positive (PRS), and random reference sets (RRS1), respectively. RLU, relative luminescence light units. (C) Histogram of the mean *Z* score values of the UPS validation set and RRS2 of interactions obtained in two independent, secondary screens. The dotted line defines the cutoff used for positive interactions. (D) Map of the NS2-UPS interactome. UPS factors are shape- and color-coded based on their function. The orange lines represent already published interactions between UPS factors; the purple lines represent interactions detected here. (E) A heat map of the positive interactions color coded based on the normalized luminescence ratio (NLR).

In search of ubiquitin signaling proteins that mediate NS2 ubiquitination, we screened for interactions between a library of the ubiquitin proteasome system (UPS) proteins and NS2 via HT-GPCA (Biquand et al., 2017). This protein-fragment complementation assay (PCA) format relies on reversible reconstitution of a luciferase activity from split *Gaussia* luciferase reporters (GLuc1 and GLuc2) and provides a high-fidelity means to measure weak and transient interactions, such as those between ESCRT and cargo (K_d_ in the μM range; Barouch-Bentov et al., 2016; Cassonnet et al., 2011; Hirano et al., 2006; Neveu et al., 2012; Raiborg et al., 2002). Moreover, it allows detection of interactions involving membrane proteins, such as NS2, in mammalian cells and within appropriate subcellular compartments (Barouch-Bentov et al., 2016; Xiao et al., 2018). The library used for this screen was recently assembled to screen for interactions with the PB2 polymerase protein of five influenza A virus strains and the E6 and E7 oncoproteins of human papilloma virus (Biquand et al., 2017; Poirson et al., 2017). The library comprises 733 cDNAs encoding 563 unique human UPS factors (Figure 1A; Table S1) and covers about one-half of the whole human UPS (Biquand et al., 2017). This library includes the UPS factors listed in two UPS-dedicated databases (Gao et al., 2013; Hutchins et al., 2013) that are available in the human ORFeome library (Rual et al., 2004) and 37 additional cDNAs encoding relevant proteins. The UPS protein-coding genes were fused to a luciferase fragment reporter (GLuc1-UPS; Biquand et al., 2017; Cassonnet et al., 2011), whereas the HCV NS2 protein, derived from the J6/JFH genome (Lindenbach et al., 2005), was fused to the complementary luciferase fragment (GLuc2-NS2). The NS2 and UPS genes were transfected pairwise into 293T cells. Luciferase activity was measured at 24 hr after transfection.

The primary screen was conducted through 10 HT-GPCA experiments, each measuring interactions of NS2 with ~80 individual UPS proteins, 11 random, a *priori* non-interacting proteins (random reference set [RRS1]), and 5 known NS2 interacting partners (positive reference set [PRS]; Barouch-Bentov et al., 2016; Cai et al., 2016; Xiao et al., 2018; Tables S2 and S3). Luminescence values were measured, and data were analyzed by boxplot. The selected positive threshold, corresponding to the third quartile plus 1.5 times the interquartile range, was validated by the distribution of the PRS and RRS1 (Figure 1B); 87 UPS factors (74 unique, 13 redundant) generated luminescence values above that threshold. To validate those hits, we retested them in triplicates, applying the NLR (normalized luminescence ratio) method to the HT-GPCA assay (Biquand et al., 2017; Table S4). We benchmarked the accuracy and sensitivity of this screen by another RRS (RRS2) composed of 53 non-interacting human protein pairs (Barouch-Bentov et al., 2016). *Z* scores, indicating the number of SDs above the mean NLR of the control RRS2, were calculated. A histogram distribution curve of the mean *Z* score values demonstrated a clear separation between the UPS validation (secondary screen) set and the RRS2 (p = 4.16 × 10^−47^, t test; Figure 1C). 38 interactions generated *Z* scores above a cutoff value of >2 SD (corresponding to an NLR of >22) in this secondary screen. Thirty of the interactors were E3 ligases (20 E3-RING; one HECT; and nine E3-CRL; Figures 1D and 1E; Table S3). Among the hits were also three E3 ligase regulators, an E2 conjugating enzyme, and four DUBs (deubiquitinating enzymes; Figures 1D and 1E).

### MARCH8 Is Required for HCV Infection

**Figure 2.**
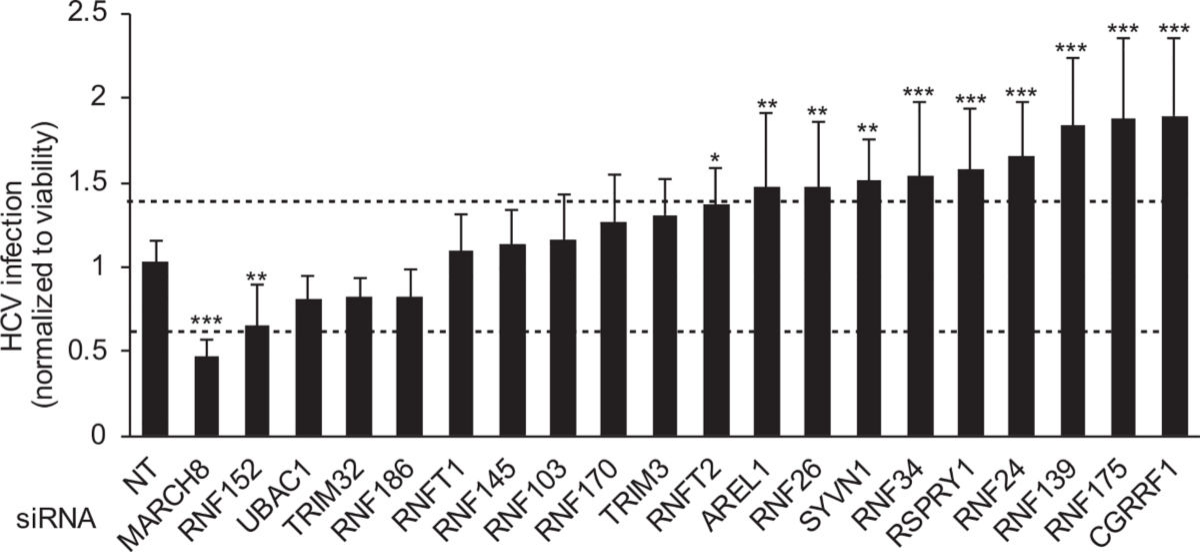
siRNA Screen Uncovers MARCH8 as an E3 Ligase Required for HCV Infection HCV (pFL-J6/JFH(p7-Rluc2A)) infection (multiplicity of infection [MOI] of 0.1) relative to NT control after siRNA-mediated knockdown of E3 ligases identified as NS2 interactors in the HT-GPCAs screen, measured via luciferase assays at 72 hr after infection in Huh7.5.1 cells and normalized to cell viability. Data are pooled from two independent experiments with 8 replicates each. Shown are means ± SD; *p < 0.05, **p < 0.01, ***p < 0.001 relative to NT control by 1-way ANOVA followed by Dunnett’s *post hoc* test.

To determine the functional relevance of E3 ligase hits to HCV infection, we examined the effects of siRNA-mediated depletion (by ON-TARGETplus SMARTpool [Dharmacon]) of 20 of those factors on HCV infection and cellular viability (Figures 2 and S1). Excluded from this screen were CYHR1 (because no SMARTpool siRNA that targets that gene is available) and the multi-subunit CRLs. Human hepatoma (Huh7.5.1) cells, depleted for the 20 E3 ligases individually or transfected with a non-targeting (NT) control siRNA, were infected with a luciferase reporter virus (pFL-J6/JFH(p7-Rluc2A); Murray et al., 2007) followed by luciferase and alamarBlue assays at 72 hr after infection. Using a cutoff of >40% inhibition of viral infection normalized to cell viability (correlates with cell density) in two independent screens, we identified MARCH8 as being required for HCV infection (Figure 2; Table S5). Although below the cutoff, depletion of RNF152 caused a statistically significant reduction in HCV infection, suggesting that RNF152 may also be required for HCV infection. In contrast, depletion of RNFT2, AREL1, RNF26, SYV1, RNF34, RSPRY1, RNF24, RNF139, RNA175, and CGRRF1 increased HCV infection by >40%, suggesting they may represent antiviral factors (Figure 2).

### NS2 Binds MARCH8 and Is Colocalized with It in the ER

**Figure 3.**
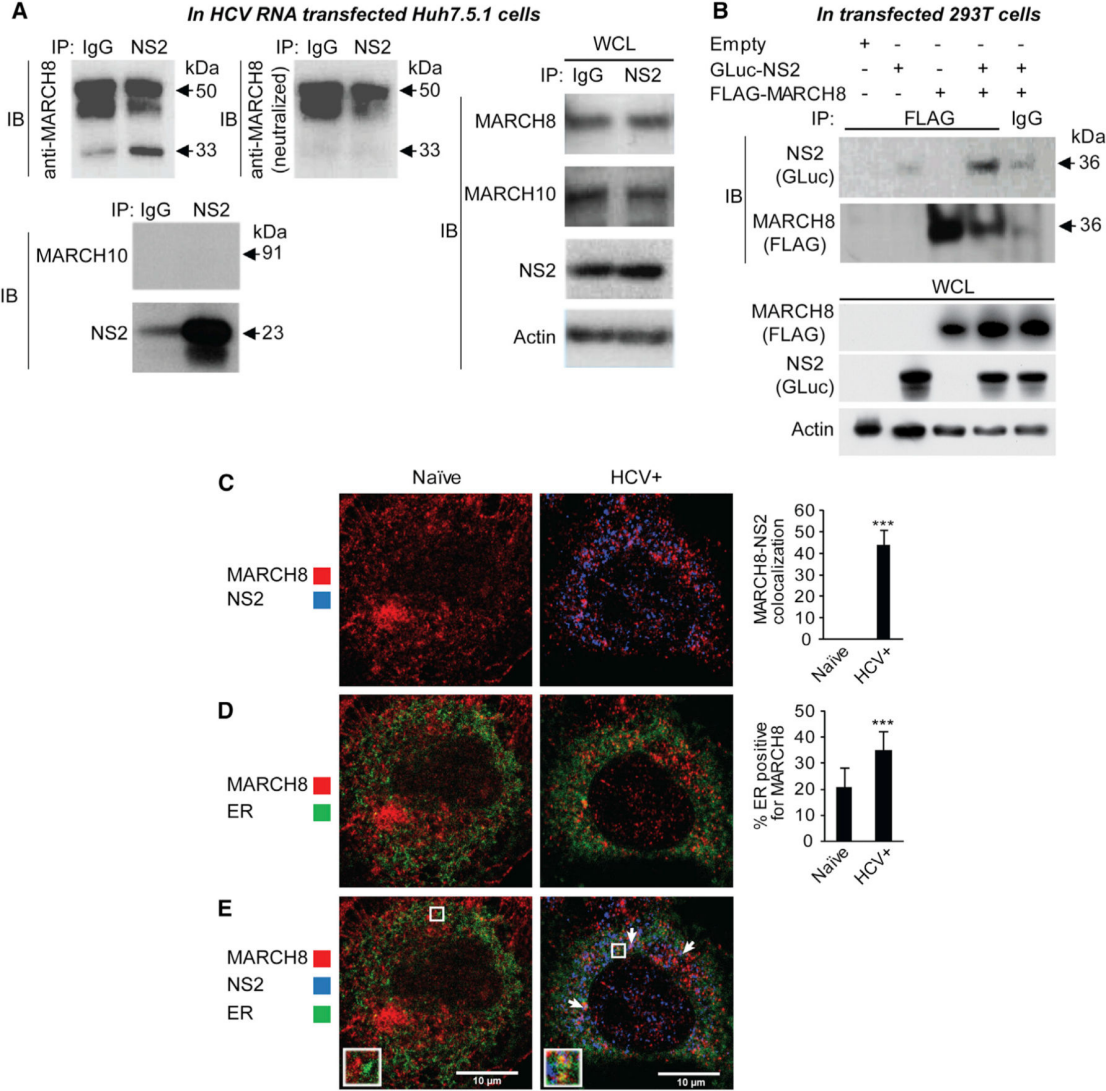
NS2 and MARCH8 Bind and Colocalize with Each Other and Are Localized to the ER (A) IPs with anti-NS2 antibody or IgG control from membrane fractions derived from Huh7.5.1 cells 72 hr after transfection with HCV RNA. Membranes were blotted with either anti-MARCH8 (top left) antibody or neutralized anti-MARCH8 antibody (after preincubation with recombinant MARCH8; top middle), and antibodies against MARCH10, NS2, and actin. Molecular weight markers are indicated on the right (kDa). Blotting with the neutralized anti-MARCH8 antibody revealed that only the 33-kDa (but not the 45- and 50-kDa) bands correspond to MARCH8. IP, immunoprecipitation; IB, immunoblotting; WCL, whole cell lysates. (B) IPs with anti-FLAG antibody or IgG control from membrane fractions derived from 293T cells ectopically expressing FLAG-MARCH8 and/or GLuc-NS2. Membranes were blotted with antibodies against GLuc, FLAG, and actin. (C–E) Confocal IF microscopy images of MARCH8 (red) and NS2 (blue) (C), MARCH8 (red) and ER (green) (D), and all three components (E) in naive and HCV (J6/JFH) RNA-transfected Huh7.5.1 cells ectopically expressing FLAG-MARCH8 48 hr after transfection. Shown are representative merged images at 60× magnification and quantitative data (means ± SD; **p < 0.01, ***p < 0.001 relative to naive control). n = ~20 cells per category. The arrows indicate MARCH8-NS2 colocalization in the ER.

Because MARCH8 emerged as an NS2 interactor that is required for HCV infection, we next validated the interaction of endogenous MARCH8 with NS2 in the context of HCV infection via co-immunoprecipitation (coIP). In membrane fractions derived from Huh7.5 cells 72 hr after transfection with HCV RNA, anti-NS2 antibody effectively pulled down NS2 (23 kDa), with which a 33-kDa protein corresponding to MARCH8 and additional (~50 kDa) protein(s) were co-immunoprecipitated (Figure 3A). Some signal was demonstrated with immunoglobulin G (IgG) control upon blotting with both anti-NS2 and MARCH8 anti-bodies, corresponding to a small fraction of NS2 that was nonspecifically pulled down with the IgG control (Figure 3A). To confirm the specificity of MARCH8 coIP, we neutralized the anti-MARCH8 antibody via preincubation with recombinant MARCH8. Blotting with this neutralized antibody eliminated the 33-kDa band but not the higher molecular weight bands, providing evidence that only the 33-kDa band corresponds to MARCH8. Lack of signal upon membrane blotting with MARCH10, another member of the MARCH family that did not bind NS2 in the PCAs screen, further confirmed the specificity of NS2-MARCH8 binding. Although reciprocal coIPs were attempted in HCV RNA transfected cells, to date none of the two anti-MARCH8 antibodies we used pulled down endogenous MACRH8. Nevertheless, anti-FLAG antibody effectively pulled down FLAG-MARCH8 ectopically expressed in 293T cells individually or with GLuc-NS2. A specific 36-kDA band, corresponding to GLuc-NS2 was co-immunoprecipitated (Figure 3B) along with MARCH8 in samples co-transfected with MARCH8 and NS2. Only a background signal was demonstrated with IgG control or in samples transfected with GLuc-NS2 alone (Figure 3B).

Next, we studied NS2-MARCH8 colocalization and the effect of HCV infection on MARCH8 subcellular localization. Because our attempts to stain endogenous MARCH8 with anti-MARCH8 antibodies were unsuccessful, we used anti-FLAG antibodies to label overexpressed FLAG-MARCH8. Significant colocalization of NS2 with MARCH8 was observed with a quantitative confocal immunofluorescence (IF) analysis of HCV RNA-transfected cells ectopically expressing FLAG-MARCH8, with Manders’ colocalization coefficients of 44% ± 6% (n = 25; Figure 3C). In mock-transfected cells, MARCH8 localized to late endosomes, as previously reported (Eyster et al., 2011; Roy et al., 2017; Samji et al., 2014), and the ER (Figure 3D). Two days after HCV RNA transfection, MARCH8 localization to the ER increased relative to control cells (Manders’ colocalization coefficients of 35% ± 7% and 21% ± 7%, respectively; n = ~20 per group; Figure 3D), whereas its localization to late endosomes did not change (Figure S2). MARCH8 did not appear to localize to lipid droplets (LD) in HCV RNA transfected or control cells (data not shown). Notably, both MARCH8 and NS2 localized to the ER, which is the presumed site of HCV envelopment (Figures 3D and 3E). Lastly, the expression level of MARCH8 did not affect the subcellular localization of NS2 (data not shown).

### MARCH8 Catalyzes K63-Linked Polyubiquitination of Lysine Residues within NS2

**Figure 4.**
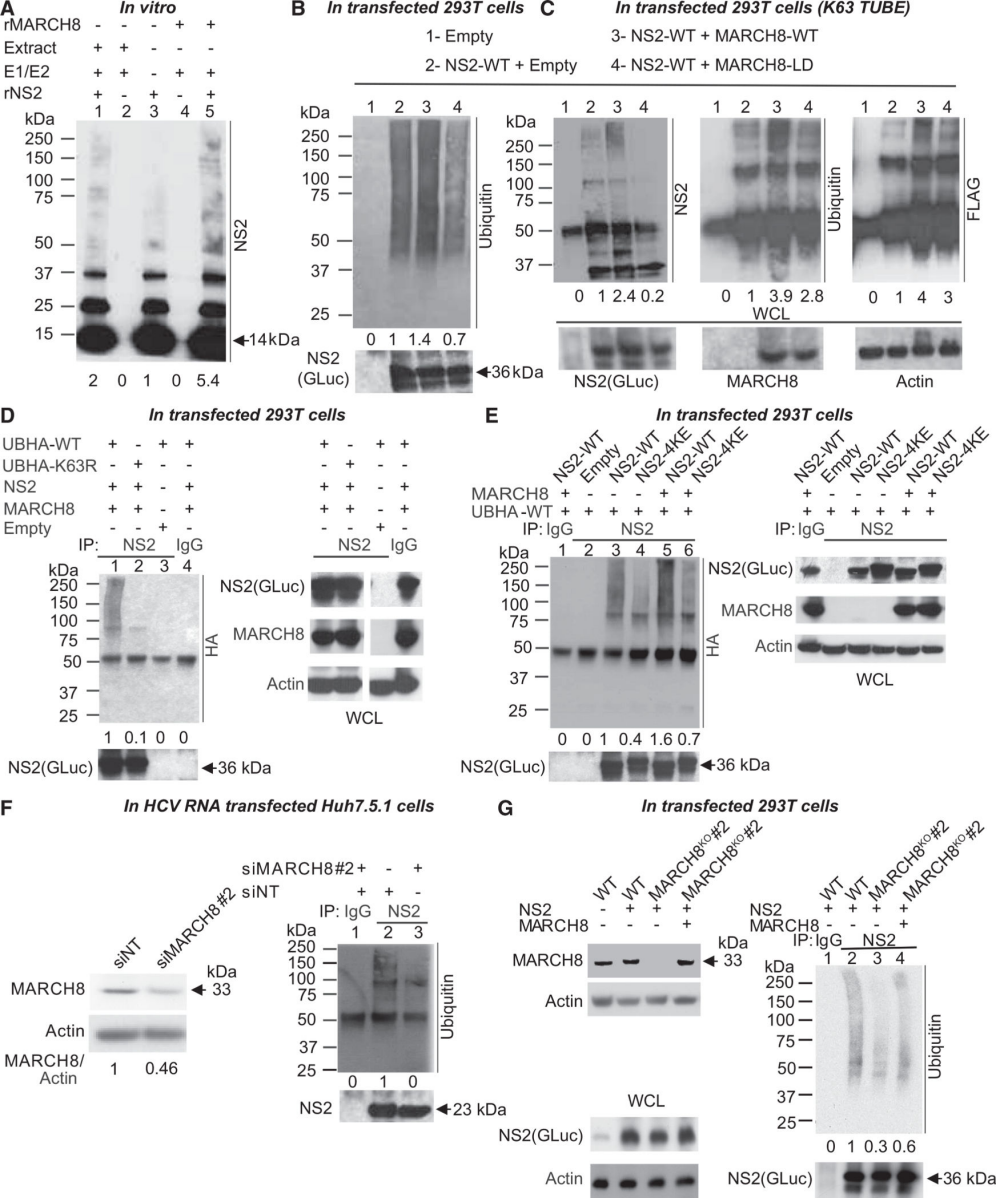
MARCH8 Ubiquitinates NS2 *In Vitro*, in Cells and in HCV RNA-Transfected Cells (A) Truncated rNS2 was incubated for 6 hr with ubiquitin alone (lane 3) or with ubiquitin, E1 activating enzyme, and UBE2H (E2 ubiquitin-conjugating enzyme) in the presence of either Huh7.5.1 cell extract (lane 1) or recombinant MARCH8 (rMARCH8; lane 5). Cell extract and MARCH8 incubated in the absence of rNS2 served as controls (lanes 2 and 4, respectively). Shown is a representative membrane blotted with anti-NS2 antibody and quantitative NS2 ubiquitination signal data relative to lane 3. (B) Lysates of 293T cells transfected with an empty plasmid (lane 1) or ectopically expressing GLuc-NS2 plus either an empty plasmid (lane 2), WT (lane 3), or ligase-dead GLuc-MARCH8 mutant (MARCH8-LD; lane 4) were subjected to IP with anti-NS2 antibody. Shown are representative membranes blotted with antibodies against ubiquitin and NS2 and quantitative data relative to lane 2. (C) Cell lysates described in (B) were incubated first with FLAG Anti-K63 TUBE reagent, followed by IP with anti-NS2 antibody. Membranes blotted with antibodies against NS2, ubiquitin, FLAG, GLuc, and actin, and quantitative data relative to lane 2 are shown. (D) Lysates of 293T cells co-transfected with UB-HA-WT (lanes 1, 3, and 4) or UB-HA-K63R mutant (lane 2), WT GLuc-NS2 (lanes 1, 2, and 4) and MARCH8 (lanes 1, 2, and 4), or empty (lane 3) plasmids were subjected to IP with anti-NS2 (lanes 1–3) or IgG (lane 4) antibodies. Membranes blotted with antibodies against HA, NS2, GLuc, MARCH8, and actin, and quantitative data relative to lane 1 are shown. WCL samples in (D) were run on the same gel, from which a few lanes were cut out. (E) Lysates of 293T cells co-transfected with UB-HA-WT (lanes 1–6), WT GLuc-NS2 (lanes 1, 3, and 5), or 4KE GLuc-NS2 mutant (NS2–4KE; lanes 4 and 6), and MARCH8 (lanes 1, 5, and 6) or empty (lanes 2, 3, and 4) plasmids, were subjected to IP with anti-NS2 (lanes 2–6) or IgG (lane 1) antibodies. Membranes blotted with antibodies against HA, NS2, GLuc, MARCH8, and actin, and quantitative data relative to lane 3 are shown. (F) Left: MARCH8 protein by western blot in cells transfected with the indicated siRNAs (numbers represent MARCH8 to actin protein ratio relative to the NT control). See Figure S3D for cellular viability data. Right: Lysates of Huh7.5.1 co-transfected with HCV RNA and the indicated siRNAs were subjected to IP with anti-NS2 (lanes 2 and 3) or IgG (lane 1) antibodies. Representative membranes blotted with anti-ubiquitin and NS2 antibodies and quantitative data relative to lane 2 are shown. (G) Left: MARCH8 protein by western blot in a 293T cell line deleted for MARCH8 via CRISPR/Cas9, this MARCH8^KO^ cell line upon ectopic expression of GLuc NS2 and/or MARCH8, and control (WT) cells. Right: Lysates of this MARCH8^KO^ cell line ectopically expressing GLuc-NS2 (lanes 1–4) and empty control (lanes 1–3) or FLAG-MARCH8 (lane 4) were subjected to IP with anti-NS2 (lanes 2–4) or IgG (lane 1) antibodies. Representative membranes blotted with antibodies against ubiquitin, GLuc, MARCH8, and actin as well as quantitative data relative to lane 2 are shown. The experiments shown in (A) were conducted three times, and those shown in (B)–(G) were conducted twice. Representative membranes are shown. The numbers below the membranes indicate the signal of NS2 ubiquitination (ladder or smear of bands >75 kDa) normalized to the respective NS2 pull down relative to the respective controls. Molecular weight markers are indicated on the left (kDa). Arrows denote molecular weights of rNS2 (A), GLuc-NS2 (B–E and G), and native NS2 (F). WCLs, whole cell lysates.

To test our hypothesis that MARCH8 mediates ubiquitination of NS2, we first conducted *in vitro* ubiquitination assays. Truncated recombinant NS2 protein (92–216 aa; rNS2), previously shown to be proteolytically active (Lorenz et al., 2006), was expressed in *E. coli* and purified (Barouch-Bentov et al., 2016). rNS2 was incubated for 2–6 hr with ubiquitin in the presence or absence of E1-activating enzyme (E1), UBE2H (an E2 ubiquitin-conjugating enzyme [E2]) that functions with MARCH8 (Goto et al., 2003; Samji et al., 2014), and either Huh7.5.1 cell extract (source of E3 ligases) or recombinant MARCH8 (rMARCH8). Samples were separated by SDS-PAGE and membranes blotted with anti-NS2 antibody. As we described (Barouch-Bentov et al., 2016), incubation of rNS2 with E1, E2, and cell extract resulted in accumulation of multiple bands (75 to >250 kDa) above the prominent bands of rNS2 (~14 kDa monomeric, 28 and 37 kDa higher orders; Figure 4A, lane 1). In the absence of E1, E2, and E3 enzymes, no such higher molecular weight band laddering was detected (Figure 4A, lane 3). A similar band pattern to that detected with cell extracts, whose intensity increased over time, was detected upon incubation of rNS2 with E1, E2, and MARCH8 (Figures 4A, lane 5, and S3A). No bands were observed when E1 and E2 were incubated with either cell extract or MARCH8 without rNS2 (Figure 4A, lanes 2 and 4). These results suggest that MARCH8 catalyzes NS2 ubiquitination *in vitro*.

Next, we studied NS2 ubiquitination by MARCH8 in 293T cells co-transfected with plasmids encoding the functionally validated GLuc-NS2 (Barouch-Bentov et al., 2016) and either MARCH8 or an empty control. Cell lysates were subjected to IP with anti-NS2 antibody. Although no bands were detected in control samples lacking NS2, samples derived from cells co-transfected with NS2 and empty control plasmid displayed a smear of bands (~45–250 kDa) that stained with anti-ubiquitin antibody, consistent with NS2 ubiquitination by endogenous E3 ligases (Figure 4B, lanes 1 and 2). The intensity of that smear of bands increased upon ectopic co-expression of NS2 and wild-type (WT) MARCH8 (Figure 4B, lane 3). Conversely, ectopic expression of MARCH10 had no effect on NS2 ubiquitination (data not shown). To test our hypothesis that MARCH8 mediates NS2 ubiquitination via its ligase activity, we introduced point mutations in three conserved residues (H107N, C110S, and W114S) within its RING-CH domain (van de Kooij et al., 2013). Co-expression of NS2 with that MARCH8-ligase-dead mutant (MARCH8-LD) reduced NS2 ubiquitination to below the basal level observed in the NS2-empty control samples, suggesting a dominant-negative effect (Figure 4B, lane 4).

We reported that NS2 undergoes K63-linked polyubiquitination (Barouch-Bentov et al., 2016). To test the hypothesis that MARCH8 mediates K63-linked NS2 polyubiquitination, samples derived from cells co-transfected with plasmids encoding GLuc-NS2 and either an empty control, WT MARCH8, or ligase-dead MARCH8 mutant were incubated for 1 hr with FLAG Anti-K63 TUBE (tandem ubiquitin binding entity; Sims et al., 2012); a reagent consisting of FLAG-tagged UIMs (ubiquitin-interacting motifs) joined by a linker that selectively binds K63-linked polyubiquitin. These samples were then subjected to IP with anti-NS2 antibody. No signal appeared in control samples lacking NS2 (Figure 4C, lane 1). NS2 ubiquitination by endogenous E3 ligases was again detected with either anti-NS2 or anti-ubiquitin antibodies in samples derived from cells expressing NS2 only (Figure 4C, lane 2). Expression of the ligase-dead MARCH8 mutant reduced NS2 ubiquitination relative to the WT MARCH8 upon blotting with an anti-ubiquitin antibody and to background level upon blotting with an anti-NS2 antibody (Figure 4C, lanes 4 versus 3). Notably, a ladder of bands with a similar molecular weight range was detected in samples expressing GLuc-NS2 upon blotting with the anti-FLAG antibody, providing evidence that the observed ubiquitination was K63 linked (Figure 4C).

To confirm that finding, lysates derived from cells co-transfected with either a WT or K63R hemagglutinin (HA)-tagged ubiquitin (UbHA) mutant, GLuc-NS2, and either MARCH8 or an empty control plasmid were subjected to IP with anti-NS2 or IgG antibodies. Ectopically expressed UbHA-K63R nearly eliminated the smear of bands detected with anti-HA antibody, relative to UbHA-WT, indicating suppression of NS2 ubiquitination by that mutant (Figure 4D, lanes 2 versus 1). No HA signal was detected in the absence of NS2 or upon pull down with IgG (Figure 4D, lanes 3 and 4). In contrast, ectopically expressed K48R mutant of UbHA did not reduce NS2 ubiquitination by MARCH8 (Figure S3B), further supporting that MARCH8 mediates K63-and not K48-linked ubiquitination of NS2.

We previously reported that mutating four lysine residues on the cytosolic surface of J6/JFH NS2 to glutamic acid (K27E-K172E-K173E-K212E; 4KE) suppresses NS2 ubiquitination (Barouch-Bentov et al., 2016). To determine whether MARCH8 mediates ubiquitination of those residues, we introduced the 4K mutations into the GLuc-NS2 vector and determined their effect on NS2 ubiquitination. 293T cells were co-transfected with either WT, 4KE NS2 mutant (NS2–4KE), or an empty plasmid, and UbHA-WT, with or without MARCH8, followed by IP with anti-NS2 antibody or IgG controls. The 4KE mutations significantly reduced the intensity of the HA-stained smear of polyubiquitination bands relative to WT NS2 in the absence of exogenous MARCH8 (Figure 4E, lanes 3 and 4) or upon ectopic expression of MARCH8 (Figure 4E, lanes 5 and 6), consistent with reduced NS2 ubiquitination. The phenotype observed with the 4KE mutant is unlikely to have resulted from an alteration in charge and/or structure because similar results were obtained when the four lysine residues were mutated to arginine (4KR; Figure S3C). These findings suggest that these NS2 lysine residues represent the acceptor sites of MARCH8-catalyzed ubiquitination, yet additional residues may be involved because residual NS2 ubiquitination was observed upon their mutation. Moreover, these findings indicate that NS2 itself, rather than the GLuc tag, undergoes ubiquitination.

We then validated the role of MARCH8 in NS2 ubiquitination in the context of HCV RNA transfection. Huh7.5.1 cells were transfected with siRNAs targeting MARCH8 and/or NT control and subsequently with J6/JFH HCV RNA. Effective depletion of MARCH8 was confirmed (Figure 4F), with no alteration in cellular viability (Figure S3D). Lysates derived from these cells were subjected to IP at 72 hr after HCV RNA transfection. NS2 antibody, but not IgG control, pulled down NS2 (Figure 4F). NT samples exhibited a ladder of bands (~75–250 kDa) after pull down with anti-NS2 antibody upon blotting with anti-ubiquitin antibody (Figure 4F, lane 2). Significant reduction in the signal >75 kDa was detected in samples derived from MARCH8-depleted cells or after IP with IgG in mixed lysates derived from cells transected with either NT or MARCH8 siRNAs (Figure 4F, lanes 3 and 1).

Because the level of siRNA-mediated NS2 suppression was modest, to further confirm the role of MARCH8 in NS2 ubiquitination, we used CRISPR-Cas9 genome engineering to generate a MARCH8-knockout (MARCH8^KO^) 293T cell line (Figure 4G). MARCH8 deficiency significantly reduced ubiquitination of ectopically expressed NS2 (Figure 4G, lanes 3 versus 2). Moreover, ectopic expression of MARCH8 in MARCH8^KO^ cells partially restored the level of NS2 ubiquitination, confirming that the reduction in NS2 ubiquitination in MARCH8^KO^ cells resulted from MARCH8 deletion (Figure 4G, lane 4).

The intensity of a nonspecific 50-kDa band appeared to change with the blocking buffer being used, from low intensity with PBS 1% casein blocker (Bio-Rad; Figures 4B, 4G, S3B, and S3C) to high intensity with milk (Figures 4C–4F). The pattern of ubiquitination (ladder versus smear >75 kDa) was variable for unknown reasons.

Notably, we measured an NS2 half-life of ~4 hr after cycloheximide-mediated blocking of protein synthesis in MARCH8-depleted and NT-control (data not shown) and MARCH8^KO^ and WT Huh7.5.1 cells (Figure S3E) transfected with HCV RNA, providing evidence that MARCH8 does not affect NS2 stability.

Together, these results indicate that the ligase activity of MARCH8 mediates K63-linked polyubiquitination of NS2 *in vitro*, in cells, and in the context of HCV infection.

### MARCH8 Is Required for HCV and DENV Assembly

**Figure 5.**
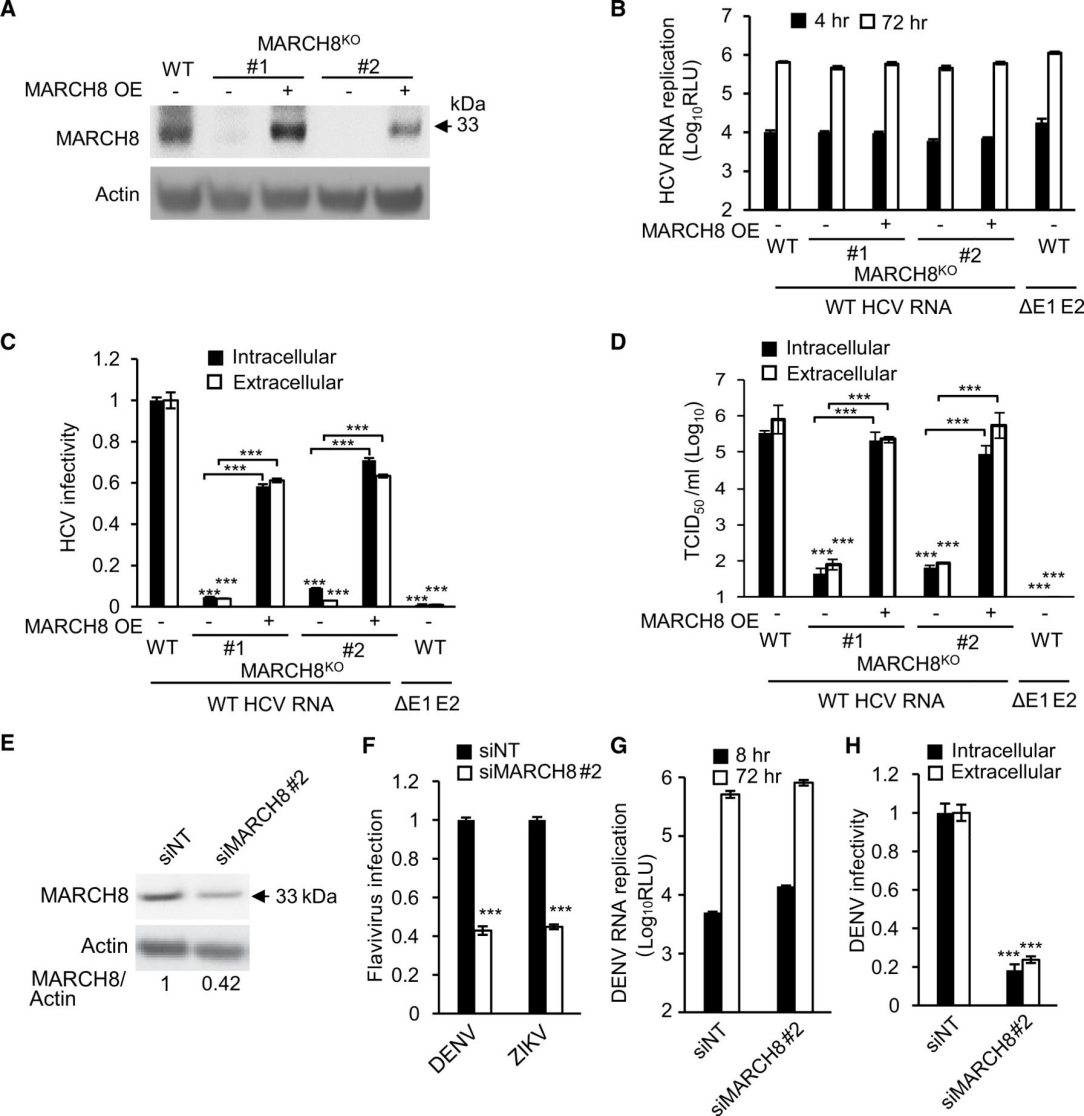
MARCH8 Is Required for HCV and DENV Assembly (A) MARCH8 protein by western blot in control Huh7.5.1 (WT) cells, two cell lines deleted for MARCH8 via CRISPR/Cas9, and these MARCH8^KO^ cell lines upon ectopic expression of MARCH8. (B) HCV RNA replication in these cells 6 and 72 hr after electroporation with WT HCV RNA or E1-E2 glycoprotein-deleted assembly defective HCV mutant (ΔE1–E2), measured by luciferase assays (RLU, relative light units; OE, overexpression). (C) HCV infectivity measured via luciferase assays by inoculating naive cells with lysates (intracellular) and supernatants (extracellular) derived from electroporated cells 72 hr after electroporation. (D) Intracellular and extracellular viral titers measured by limiting dilution assays. TCID_50_, 50% tissue culture infectious dose. (E) MARCH8 protein in Huh7 cells transfected with the indicated siRNAs (numbers represent MARCH8-to-actin protein ratio relative to the NT control). (F) DENV2 (MOI = 0.1) and ZIKV (MOI = 0.05) infection in MARCH8-depleted cells, measured by luciferase assays at 48 and 72 hr, respectively, and normalized to cell viability. (G) DENV2 RNA replication in MARCH8-depleted Huh7 cells measured by luciferase assays at 8 and 72 hr after electroporation with DENV RNA. (H) DENV2 infectivity measured via luciferase assays by inoculating naive cells with lysates (intracellular) and supernatants (extracellular) from electroporated cells. (B)–(D) represent data pooled from three independent experiments each with 3–6 biological replicates. (F)–(H) are representative experiments out of two conducted. Shown are means ± SD; ***p < 0.001 relative to corresponding WT or NT controls by one-way (C, D, F, and H) or two-way (B and G) ANOVA with Dunnett’s (F, G, and H) or Tukey’s (B, C, and D) *post hoc* tests.

To pinpoint the stage in the life cycle that MARCH8 is involved in mediating, we generated two isogenic MARCH8^KO^ Huh7.5.1 cell lines using CRISPR/Cas9 (Figure 5A). To minimize any potential off-target effects of Cas9, we performed subsequent analyses with these two independently generated KO clonal cell lines. MARCH8 KO had no effect on HCV RNA replication, as measured by luciferase assays at 4, 24, 48, and 72 hr after transfection with J6/JFH *Renilla* reporter RNA (Murray et al., 2007; Figures 5B, S4A, and S4B). Inoculation of naive cells with clarified cell lysates or culture supernatants derived from the HCV RNA transfected cells at various times after transection, followed by luciferase assays at 72 hr, revealed a >1-log reduction in both intracellular and extracellular infectivity at 72 hr after electroporation, respectively, indicating a defect in HCV assembly (Figures 5C, S4C, and S4D). Consistent with those data, MARCH8^KO^ cells exhibited a >3-log reduction in the intracellular and extracellular HCV titer measured via limiting-dilution assays (Figure 5D). Transfection of the envelopment-defective HCV RNA deleted for the E1 and E2 glycoproteins (ΔE1–E2) produced luciferase signals at the background level and entirely suppressed viral titer (Figures 5C and 5D). Ectopic expression of MARCH8 in the two MARCH8^KO^ cell lines either partially or completely restored the level of infectivity measured via luciferase and limiting-dilution assays, confirming that the defect detected in MARCH8^KO^ cells resulted from MARCH8 deletion (Figures 5C and 5D).

Reduced levels of HCV RNA and core protein release were measured in culture supernatants of MARCH8^KO^ cells relative to WT cells by qRT-PCR and ELISA assays, respectively, whereas the intracellular core level was not altered (Figures S4E–S4G). Nevertheless, the level of defective, noninfectious particles released by MARCH8 deletion was not greater than that released by the assembly-defective ΔE1–E2 mutant. Similar to the ΔE1–E2 mutant, MARCH8 deletion significantly reduced the specific infectivity (the ratio of focus-forming unit per viral RNA molecules) relative to WT cells (Figure S4G). Taken together, these results provide evidence that MARCH8 deletion disrupts HCV assembly.

A similar effect on HCV assembly was demonstrated in cells transiently depleted of MARCH8 by two siRNAs (Figures S4H–S4L). Ectopic expression of siRNA-resistant MARCH8 reversed the HCV assembly defect induced by MARCH8 depletion (Figures S4M and S4N), largely excluding the possibility of off-target effects causing the observed phenotype.

To investigate whether distantly related members of the *Flaviviridae* family rely on MARCH8, we examined the effect of MARCH8 gene silencing on infection with the luciferase reporter DENV2 New Guinea C (NGC) strain (Xie et al., 2013; Zou et al., 2011) and ZIKV (Asian strain; Shan et al., 2016). MARCH8 siRNA-mediated knockdown in Huh7 cells suppressed DENV and ZIKV infections (Figures 5E and 5F) with no apparent effect on cellular viability (Figure S4O; correlates with cell density; data not shown). Moreover, depletion of MARCH8 followed by transfection with NGC luciferase reporter RNA had no effect on DENV2 RNA replication (Figure 5G), but analogous to experiments with HCV, it diminished the production of infectious virus in cell lysates and culture supernatants relative to NT controls (Figure 5H). These results implicate MARCH8 in several *Flaviviridae* infections and in assembly of both HCV and DENV.

### The Ligase Activity of MARCH8 Is Required for HCV Assembly

**Figure 6.**
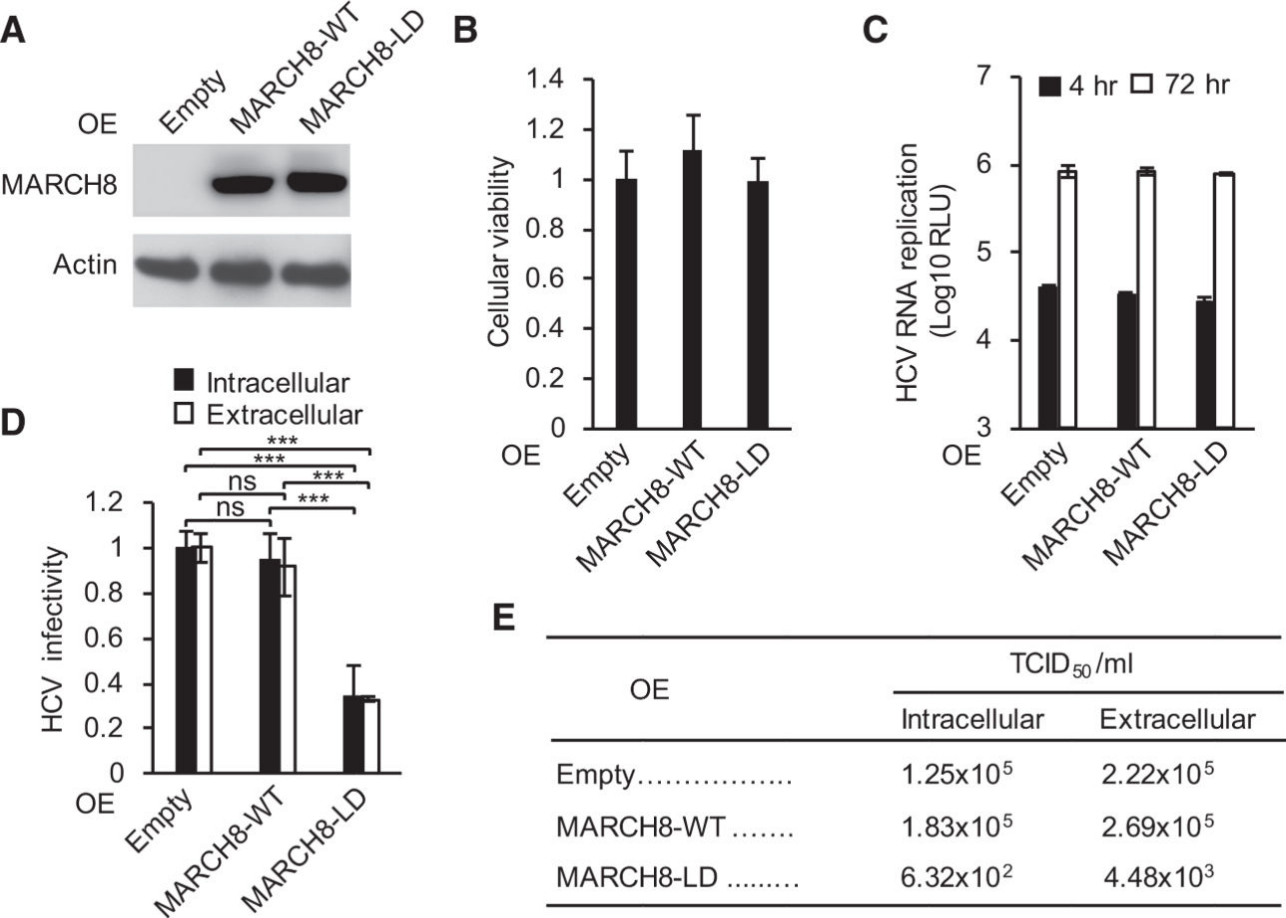
MARCH8 Ligase-Dead Mutant Disrupts HCV Assembly (A and B) MARCH8 protein (A) or relative cell viability (B) in cells ectopically expressing MARCH8 or empty plasmid control. LD, ligase dead. (C) HCV RNA replication in cells transfected with the indicated plasmids 4 and 72 hr after electroporation with WT HCV RNA measured by luciferase assays (RLU, relative light units). (D) and E) Intracellular and extracellular infectivity measured by luciferase assays (D) and limiting dilution assays (E) in naive cells inoculated with clarified cell lysates or supernatants derived from the HCV electroporated cell lines, respectively. TCID_50_, 50% tissue culture infectious dose. (B)–(D) represent data pooled from three independent experiments each with 3–6 biological replicates. (E) is a representative experiment out of two conducted. Shown are means ± SD; ***p < 0.001 relative to empty plasmid control by one-way (B and D) or two-way (C) ANOVA with Dunnett’s (B and C) or Tukey’s (D) *post hoc* tests.

To determine the role of MARCH8’s ligase activity in HCV assembly, we used a transdominant interfering approach. Ectopic expression of WT or ligase-dead MARCH8 mutant had no effect on cellular viability or HCV RNA replication at 4 and 72 hr after electroporation with J6/JFH(p7-Rluc2A) RNA (Figures 6A–6C). Ectopic expression of the ligase-dead MARCH8 mutant, but not the WT MARCH8, significantly reduced both the intracellular and extracellular infectivity relative to an empty control plasmid, as measured by luciferase and limiting-dilution assays (Figures 6D and 6E). These results indicate that an intact ligase function of MARCH8 is required to facilitate its role in HCV assembly and that its endogenous expression level is likely not rate limiting for HCV infection.

### MARCH8 Mediates HCV Envelopment

**Figure 7.**
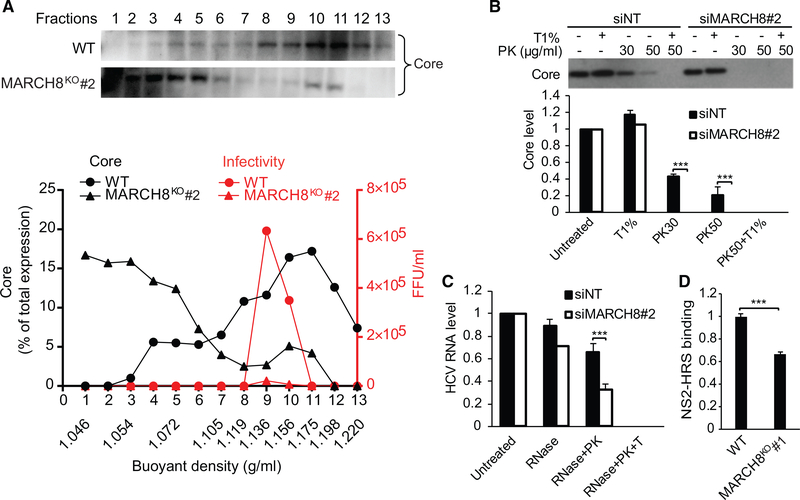
MARCH8 Mediates HCV Envelopment (A) Sucrose density-gradient analysis of intracellular viral particles derived from control (WT) and MARCH8^KO^ cells 3 days after transfection with HCV RNA by isopycnic separation (see Figure 5A for protein expression). Thirteen fractions were collected and analyzed. The experiment was conducted twice. Representative membranes blotted with anti-core antibody are shown. Plotted are the core content along the gradient normalized to the total core protein (black) and levels of infectivity (focus-forming units [FFU] per milliliter; red). (B) Proteolytic digestion protection assays in lysates of HCV RNA transfected cells depleted of MARCH8 or NT controls (see Figure 4F for protein expression). Shown are representative membranes and quantitative data pooled from two independent experiments demonstrating the level of protease-resistant core after no treatment or treatment with Triton (T) and/or PK. (C) Fraction 6 of a rate zonal-like gradient was subjected to RNase digestion protection assays. Samples were left untreated or treated with the S7 RNase either alone, after PK, or after Triton and PK. Plotted is residual HCV RNA measured by qRT-PCR assays pooled from two experiments. (D) NS2-HRS binding in MARCH8^KO^ and WT cells measured via PCAs pooled from two experiments. Shown are means ± SD; ***p < 0.001 relative to the corresponding untreated or WT control by one-way ANOVA with Tukey’s *post hoc* test (C) and Student’s t test (D).

To pinpoint the precise role of MARCH8 in HCV assembly, we first characterized the density of intracellular viral particles via isopycnic separation. Clarified lysates of MARCH8^KO^ cells (see Figure 5A for protein expression) transfected with HCV RNA or control (WT) cells were layered on top of a sucrose gradient (10%–60%) and spun for 16 hr. Thirteen fractions were collected and subjected to measurement of buoyant density by a refractometer, core levels by immunoblotting, infectivity by focus formation assays, and HCV RNA by qRT-PCR. A prominent peak of core was observed in fractions 9–11, which coincided with the peak of infectivity in WT samples (Figure 7A). The density of intracellular particles in fractions harboring the peak of the core in WT samples was 1.156–1.175 g/mL, as previously reported (Barouch-Bentov et al., 2016). MARCH8 deficiency both suppressed infectivity and shifted the distribution of the core to fractions 1–5 of the gradient (Figure 7A). These data suggest that fractions 9 and 10 carry the bulk of the infectious particles, whereas the core protein species sedimenting in fractions 1–5 likely represent non-enveloped particles. MARCH8 absence may thus increase accumulation of non-enveloped nucleocapsids.

To further characterize this phenotype, we monitored envelope protection of the capsid by examining the resistance of core to proteinase K (PK) digestion. Lysates derived from MARCH8 siRNA-depleted or NT control cells (Figure S4H) transfected with J6/JFH HCV RNA were left untreated or treated with 1% Triton and/or PK. Residual core protein was quantified by western blotting. Although treatment with Triton alone did not affect core abundance, PK alone resulted in core proteolysis in the NT control samples (Figure 7B). Nevertheless, a fraction of core remained protected from the protease in the NT samples, consistent with an intact envelope. As predicted, the core protein underwent complete proteolysis by PK after pretreatment with Triton in NT samples. In lysates derived from MARCH8-depleted cells, the core protein was unaffected by Triton treatment alone; however, even a low concentration of PK alone was sufficient to completely degrade it (Figure 7B).

To characterize the fate of HCV RNAs in MARCH8-depleted cells, we examined their sensitivity to RNase digestion. To do so, clarified lysates of HCV-transfected, MARCH8-depleted or NT control cells were layered on top of a sucrose density gradient (5%–35%) and spun for 1 hr. These hybrid separations performed much like rate zonal gradients (Barouch-Bentov et al., 2016). Ten fractions were collected, and the infectivity and refraction index were measured by focus formation assays and a refractometer, respectively. Although absolutely no infectivity was detected in MARCH8-depleted samples in any of the fractions (data not shown), as reported (Barouch-Bentov et al., 2016; Gentzsch et al., 2013), the peak of infectivity in NT samples was in fractions 6 and 7 (with refraction indices of 1.362 and 1.367, corresponding to densities of 1.075 and 1.088 g/mL, respectively). Fraction 6 of the gradient was thus subject to RNase digestion. As previously described (Barouch-Bentov et al., 2016; Gentzsch et al., 2013), samples were left untreated or subjected to treatment with the S7 RNase either alone, after PK, or after PK plus Triton. Residual HCV RNA was quantified by qPCR. Viral RNA in the NT samples was protected from RNase-only treatment and RNase after pretreatment with PK and was fully susceptible to RNase after pretreatment with Triton and PK (Figure 7C). HCV RNA was similarly protected in MARCH8-depleted samples when RNase was used alone; however, there was a significant reduction in the protected viral RNA fraction upon pretreatment with PK (Figure 7C). HCV RNAs were fully susceptibility to RNase in MARCH8-depleted samples pretreated with both Triton and PK.

Together, these data indicate that MARCH8 contributes to the formation of a proteinaceous and membranous shell capable of protecting the viral genome from degradation in the absence of detergents rather than a post-budding step critical for infectivity. Although HCV RNA replication complexes are also capable of protecting the genome from nucleases (Miyanari et al., 2003; Quinkert et al., 2005), because our data indicate that MARCH8 is not involved in HCV RNA replication, we favor the explanation that MARCH8 contributes to the envelopment of HCV particles.

Lastly, we studied binding of NS2 to its ESCRT-interacting partner, HRS (Barouch-Bentov et al., 2016), in the MARCH8^KO^ cell line by PCAs. MARCH8 deletion moderately reduced NS2-HRS binding (Figure 7D), supporting the idea that MARCH8 mediates its role in HCV envelopment at least in part by catalyzing NS2 ubiquitination and, subsequently, HRS recruitment. The detected residual HRS binding suggests that additional E3 ligases may be involved in mediating K63-linked polyubiquitination of NS2.

## DISCUSSION

We reported that K63 polyubiquitination of lysine residues within NS2 mediates its binding to HRS, an ESCRT-0 complex component, and that this interaction is essential for HCV envelopment (Barouch-Bentov et al., 2016). Nevertheless, the upstream ubiquitin signaling factors that govern NS2 ubiquitination and HCV envelopment as well as the precise E3 ubiquitin ligase remained unknown. By integrating proteomic, genomic, transdominant interference and biochemical approaches, we map the interaction network of NS2 with ubiquitin signaling factors and reveal candidate proviral and antiviral interactors. We provide evidence that MARCH8, a RING-type E3 ligase previously shown to catalyze K48-linked ubiquitination of host substrates, mediates HCV NS2 K63 polyubiquitination. Moreover, we demonstrate that the E3 ligase activity of MARCH8 is essential for NS2 ubiquitination and HCV envelopment. These findings provide insights into the virus-host determinants that regulate HCV envelopment, shed light on the function of an understudied E3 ubiquitin ligase, and have potential implications for the design of antiviral strategies.

Our screens discovered ubiquitin signaling factors that govern NS2 ubiquitination. Thirty interactions between NS2 and E3 ligases were identified in our proteomic screen, 20 of which involved ligases from the class of non-cullin RING E3 ligases. Notably, none of these E3 ligases overlapped with those identified as partners of the influenza A virus BP2 polymerase protein and the two human papillomavirus oncoproteins when screened against the same library by the same screening approach (Biquand et al., 2017; Poirson et al., 2017). This differential binding highlights the specificity of the HT-GPCA platform and indicates that the identification of multiple E3 ligases as NS2 partners likely reflects the high sensitivity of this platform and the nature of E3 ligases (which represent the largest fraction of the human UPS and the main UPS interactors of viral proteins). Because both NS2 and MARCH8 are transmembrane proteins, these studies also demonstrate the utility of this platform to measure difficult-to-study protein interactions. Twelve E3 ligases identified as NS2 interactors, including MARCH8, were found to be potentially functionally relevant to HCV infection. Knockdown of those E3 ligases had variable effects ranging from >40% reduction to >40% increase in viral infection, suggesting that they may act as proviral or antiviral factors, respectively, and may have diverse roles in HCV infection. Future work is required, however, to further validate their role in HCV infection.

Our results indicate that MARCH8 binds and ubiquitinates NS2. MARCH8 was previously shown to mediate K48-linked ubiquitination and degradation of the cellular protein ILRAP1 (Chen et al., 2012). Our finding that it also catalyzes K63 polyubiquitination has not been previously reported and may contribute to better understanding the roles of MARCH8 in cellular processes beyond proteasomal degradation, such as its activity in endosomal sorting (Eyster et al., 2011). Generation of different ubiquitin linkages by the same RING-type E3 ligase (e.g., K48 and K63) was previously reported and, in some cases, is thought to be dependent on the specific E2 ligase with which they are paired (Chastagner et al., 2006; Lee et al., 2008; Metzger et al., 2014). Interestingly, K3, the Kaposi’s sarcoma-associated herpesvirus RING-CH E3 ligase, which MARCH8 was originally identified as a cellular homolog of (Goto et al., 2003), mediates K63-linked polyubiquitination (Duncan et al., 2006). Nevertheless, ubiquitination catalyzed by MARCH8 and various herpes viral RING-CH E3 ligases downregulates receptors, such as MHC-II proteins and interleukin-1 (IL-1) receptor, thereby promoting viral immune evasion (Randow and Lehner, 2009). Our data indicate that viruses from the *Flaviviridae* family that do not encode a RING-CH E3 ligase, hijack MARCH8 to mediate a different role in viral infection beyond the previously reported role in immune regulation, i.e., viral assembly.

To date, members of the MARCH family have been implicated in ubiquitination of host proteins. Although a functional interaction between MARCH8 and HIV-1 has been reported (Tada et al., 2015), no viral protein has been previously shown to be ubiquitinated by MARCH8. The discovery that NS2 functions as a ubiquitination substrate for the ligase activity of MARCH8 thus provides insights into virus-host interactions with potential implications for other viruses.

We previously reported that cytoplasmic lysine residues within NS2 undergo K63-linked ubiquitination, which mediates HRS UIM binding and, subsequently, HCV assembly and that HRS mediates HCV envelopment (Barouch-Bentov et al., 2016). Our current mechanistic studies indicate that MARCH8 is an E3 ligase mediating this K63-linked polyubiquitination of NS2 and, subsequently, HRS binding and HCV envelopment. Intriguingly, MARCH8 has been implicated in regulation of ESCRT-mediated endosomal sorting. MARCH8 is detected in endosomal compartments co-stained with HRS and CD63, and its depletion increases the localization of BST2, a ubiquitination substrate, to the HRS-positive, late-endosomal compartment (Eyster et al., 2011; Roy et al., 2017; Samji et al., 2014). Moreover, transport of various host cargo proteins by the ESCRT machinery is dependent on MARCH8 ubiquitination (Eyster et al., 2011). Our findings thus propose a role for MARCH8 in endosomal sorting of a viral protein substrate and suggest that MARCH8-catalyzed K63-linked ubiquitination may also be involved in ESCRT-mediated sorting of host cargo proteins.

Combined with our reported data (Barouch-Bentov et al., 2016), our findings suggest that MARCH8-mediated NS2 ubiquitination regulates HCV envelopment. Notably, other E3 ligases are implicated in envelopment of viruses at the plasma membrane. Specifically, Nedd4 E3 ligases interact with the late-domain motif PPXY within viral structural proteins and mediate their interactions with ESCRT components, subsequently leading to viral envelopment (Martin-Serrano et al., 2005). Similarly, the HIV Gag protein, which lacks a PPXY domain, recruits a Nedd4-like E3 ligase to ubiquitinate ESCRT components, activates them, and facilitates viral envelopment (Chung et al., 2008; Usami et al., 2008). We show that HCV, which lacks defined late-domain motifs, uses a cellular E3 ligase to ubiquitinate a viral protein to facilitate envelopment. Moreover, although the Nedd4 family and Nedd4-like family are members of the HECT class of E3 ligases, MARCH8 belongs to the RING finger class of E3 ligases. Our data thus reveal a ubiquitin signaling mechanism used by viruses lacking late-domain signals to ubiquitinate a viral protein. By demonstrating that this ubiquitination then serves as the signal to recruit the ESCRT machinery and to facilitate intracellular viral envelopment (Barouch-Bentov et al., 2016), we reveal a role for ubiquitin signaling in HCV infection beyond regulation of viral protein stability.

It is possible that, similar to other substrates (Metzger et al., 2014), NS2 is ubiquitinated by additional E3 ligases. Future studies will determine the roles of other E3 ligases identified in our screens in NS2 ubiquitination. It is also possible that a number of E3 ligases differentially regulate various NS2 activities. Indeed, ubiquitination is one of the most versatile post-translational modifications and is a highly regulated process. It may, therefore, provide an effective means of tightly regulating the activities of a viral protein, such as NS2, which is involved in multiple steps in the HCV viral life cycle. The interactions of NS2 with DUBs and E3 ligase regulators support hijacking of various aspects of ubiquitin signaling for tight regulation of NS2 functions and represent another area of future investigation.

Viruses co-opt the UPS to manipulate the host cell cycle, membrane trafficking, DNA repair, or apoptosis as well as to evade the immune system and exit the infected cell (Randow and Lehner, 2009). E3 ligases represent attractive, druggable molecular targets for pharmacological inhibition. Indeed, the use of bortezomib, a proteasome inhibitor, for the treatment of cancer illustrates that potential (Citrin et al., 2016). Although still in their infancy, selective inhibitors of E3 ligases have also shown promise as potential anticancer drugs (Sun, 2003). Such compounds are likely to achieve a high level of substrate specificity, thereby reducing toxicity (Sun, 2003). Selective targeting of E3 ligases could thus represent an attractive antiviral strategy. Our data showing that MARCH8 is also required for DENV and ZIKV infections, suggest that MARCH8 may represent a target for broader-spectrum antivirals, similar to cellular kinases (Bekerman and Einav, 2015; Bekerman et al., 2017). The precise role of MARCH8 in these viral infections will be studied in the future.

Taken together, these results validate virus-host interactions required for intracellular HCV envelopment, reveal a role for MARCH8 in K63-linked ubiquitination of a viral protein substrate and HCV envelopment, and propose a role for MARCH8 in DENV assembly. These findings may have implications for the roles of MARCH8 in cellular processes and other viral infections and for the design of host-targeted antiviral strategies.

## STAR★METHODS

### CONTACT FOR REAGENT AND RESOURCE SHARING

Further information and requests for resources and reagents should be directed to and will be fulfilled by the Lead Contact, Shirit Einav (seinav@stanford.edu). An approved Material Transfer Agreement (MTA) may be required for resource sharing.

### EXPERIMENTAL MODEL AND SUBJECT DETAILS

#### Cell lines

Huh7.5.1 (Apath LLC), Huh7 (Apath LLC) and 293T (ATCC) cells were grown in DMEM supplemented with 10% fetal bovine serum (Omega Scientific), 1X nonessential amino acids, 1% L-glutamine and 1% penicillin-streptomycin (GIBCO).

### METHOD DETAILS

#### Plasmids

ORFs encoding the UPS library were selected from the Human ORFeome library of cDNA clones (Open biosystems) (Rual et al., 2004) and recombined into pGLuc or pFLAG vectors by Gateway technology (Invitrogen). ORF encoding HCV NS2 was amplified from described vectors (Murray et al., 2007) and recombined into pGLuc vector. pFL-J6/JFH(p7-Rluc2A) *Renilla* reporter HCV plasmid was a gift from Dr. Rice (Murray et al., 2007). DENV2 (New Guinea C strain) (Xie et al., 2013; Zou et al., 2011) and ZIKV (FSS13025, Asian strain) (Shan et al., 2016) *Renilla* reporter plasmids were gifts from Pei-Yong Shi. siRNA-resistant MARCH8 was cloned by introducing a wobble mutation in the siRNA-targeted site. This mutation, the MARCH8 ligase-dead mutation, and NS2 lysine mutations were introduced by the QuikChange kit (Stratagene). Primer sequences will be provided upon request.

#### Gaussia split-luciferase protein-fragment complementation assays (GPCAs)

The primary screen was conducted by co-transfecting combinations of plasmids encoding prey (A) and bait (B) proteins, each fused to a fragment of the Gaussia luciferase protein (GLuc1 and GLuc2) into 293T cells plated in 96-well plates in triplicates. At 24 hours post-transfection, cells were lysed and subjected to luciferase assays (Promega). Results were expressed as RLU. The secondary screen was conducted as above, except that control plasmids were also transfected with the individual ORFs, as described (Cassonnet et al., 2011; Neveu et al., 2012), and results were expressed as NLR: the average signal in cells transfected with GLuc1-A and GLuc2-B divided by the average signal in wells transfected with GLuc1-A and an empty GLuc2 plasmid and those transfected with GLuc2-B and an empty GLuc1 plasmid.

#### RNA interference

Custom Cherry-Pick ON-TARGETplus SMART pools siRNA library against E3 ligase genes and a non-targeting control (D-001810–10-05) were purchased from Dharmacon (Table S5). Silencer select siRNAs targeting MARCH8 [s47920 (GGACATTTCATGAGT CATT), s47921 (GGAAGAGACTCAAGGCCTA)] and a non-targeting control (4390843) were from Life Technologies. siRNAs (100–200 nM) were transfected into Huh7.5.1 or Huh7 cells using silMPORTER (Millipore) 72 hours before infection and 50 nM were boosted concurrently with HCV or DENV transfection.

#### Co-immunoprecipitations

Co-IPs in membrane fractions derived from Huh7.5.1 cells transfected with HCV RNA or from CRISPR/Cas9 MARCH8^KO^ cells transfected with FLAG-MARCH8 and/or GLuc-NS2 were carried out, as described (Neveu et al., 2012). ~20 × 10^6^ Huh7.5.1 cells were collected by trypsinization, washed with PBS and incubated with 1mM dithiobis-succinimidyl-propionate (DSP) crosslinker (Pierce) solution for 2 hours on ice (to allow covalent binding of the already bound interacting proteins). Tris (pH 7.5) was added at 20 mM for 15 minutes to quench unreacted DSP. Cells were washed once with PBS, resuspended in HME buffer (20 mM HEPES (pH 7.4), 1 mM EDTA, 2 mM MgCl2) supplemented with phenylmethylsulfonyl fluoride to a final concentration of 1 mM and a protease inhibitors cocktail (Sigma). Cells were lysed by two freeze-thaw cycles and passaged through a 27.5-gauge needle 10 times. Nuclei were removed by centrifugation at 250 × g for 10 minutes, and the postnuclear supernatant was subjected to ultracentrifugation at 100,000 × g for 30 minutes. All steps were done at 4°C. Membrane pellets were resuspended in 100 μl HME buffer. TDB buffer (2.5% Triton X-100, 25 mM triethanolamine-Cl (pH 8.6), 20 mM NaCl, 5 mM EDTA, 0.2% NaN3) was added to a final volume of 1 ml. Samples were incubated with anti-NS2 antibody for ~2 hours and then overnight with protein A/G magnetic beads (Dynabeads, Life Technologies). Following PBS washes, bound proteins were eluted in SDS sample buffer.

#### IF confocal microscopy

IF was performed in Huh7.5.1 cells 48 hours post-transfection with HCV J6/JFH RNA and a FLAG-MARCH8 plasmid, as described (Neveu et al., 2012). For ER labeling, CellLight® ER-GFP, BacMam 2.0 (Invitrogen) was used to transduce cells 32 hours postelectroporation of HCV RNA and incubated for 16 hours prior to IF staining. Late endosomes were labeled via transfection of a plasmid encoding RAB7-GFP (a gift from Dr. Pfeffer, Stanford). LD staining with Bodipy-488/503 (Invitrogen) was performed as described (Neveu et al., 2012). Colocalization was quantified in ~20 randomly chosen cells from each sample using ImageJ (JACoP) colocalization Software and Manders’ Colocalization Coefficients (MCC).

#### Infection assays

Huh7.5.1 were infected with HCV pFL-J6/JFH(p7-Rluc2A) and Huh7 cells with DENV2 or ZIKV *Renilla* reporter plasmids in 8–12 replicates for 4 hours at an MOI of 0.1 (HCV and DENV) and 0.05 (ZIKV). Overall infection was measured at 72 (HCV, ZIKV) or 48 (DENV) hours using standard luciferase assays.

#### *In vitro* transcription of viral RNA and transfection

HCV or DENV RNA was generated and delivered into Huh-7.5.1 cells, as previously described (Lindenbach et al., 2006). Briefly, RNA was synthesized from XbaI linearized J6/JFH (p7-Rluc2A) template using the T7 MEGAscript kit according to the manufacturer’s protocol (Ambion). Reaction mixtures were incubated for 3 hr at 37°C and then subjected to DNase treatment for 15 min at 37°C. Viral RNA was purified using the RNeasy kit (QIAGEN). RNA was quantified by absorbance at 260 nm, and its quality was verified by agarose gel electrophoresis. Subconfluent Huh-7.5.1 cells were trypsinized and collected by centrifugation at 700 g for 5 min. The cells were then washed three times in ice-cold RNase-free PBS (BioWhittaker) and resuspended at 1.5*10^7^ cells/ml in PBS. 6 μg of the *in vitro* transcribed wild-type or J6/JFH(p7-Rluc2A) mutant RNA was mixed with 400 μl of washed Huh-7.5.1 cells in a 2 mm-gap cuvette (BTX) and immediately pulsed (0.82 kV, five 99 μs pulses) with a BTX-830 electroporator. After a 15 min recovery at 25°C, cells were diluted in 30 mL of prewarmed growth medium and plated into 96, 24, 6-well and P100 tissue culture plates.

#### HCV and DENV RNA replication by luciferase assays

HCV and DENV RNA replication was measured at 6–9 hr and 72 hr postelectroporation, as described (Bekerman et al., 2017; Murray et al., 2007). Electroporated cells plated in quadruplicates in 96-well plates were washed twice with PBS and lysed with 50 μl of *Renilla* lysis buffer (Promega). Following 15 min of shaking at RT, luciferase activity was quantified using a *Renilla* luciferase substrate (Promega) and a Tecan luminometer (Tecan) according to the manufacturers’ protocols.

#### Extracellular infectivity

Culture supernatants of cells electroporated with HCV or DENV RNA and plated in P100 dishes were harvested at 72 hr postelectroporation, clarified (with a 0.22-μm-pore size filter) and used to infect naive cells for 72 hr in triplicates before lysis in *Renilla* lysis buffer (Promega). Luciferase activity in 20 μl of cell lysates was quantified as described above. Results represent log10 RLU values per 10 cm tissue culture dish.

#### Intracellular infectivity assays

72 hr postelectroporation with HCV or DENV RNA, cells were trypsinized, collected by centrifugation, resuspended in 500 μl medium, lysed by freeze-thaw cycles, and pelleted twice at 3,650 × g. Clarified supernatants diluted in complete medium were used to inoculate naive cells in triplicates, followed by lysis and luciferase assays at 72 hr. Results represent log10 RLU values per 10 cm tissue culture dish.

#### Virus titration

Extracellular and Intracellular titers were determined by limiting dilution assays based on immunohistochemical staining for core. 50% tissue culture infectious dose (TCID_50_) was calculated, as described Lindenbach et al., 2005. Results are expressed as TCID_50_/ml. Minimal titers measured with the ΔE1–E2 mutant were used for background subtraction.

#### RNA extraction and qRT-PCR

Total RNA was isolated from cells, cell culture supernatants or gradient fractions using TRIzol (Invitrogen) or NucleoSpin RNA virus kit (Macherey-Nagel). qRT-PCRs mixtures were assembled in triplicates using High-Capacity RNA-to-cDNA and Power SYBR Green RNA-to-CT 1-Step Kit (Cat No:4389986: Applied Biosystems). The primers are listed in Table S6. Amplification and analysis were performed using StepOnePlus Real-Time-PCR system (Applied Biosystems). GAPDH was used as a control.

#### Generation of MARCH8 knockout cell lines

CRISPR guide RNA (gRNA) sequences were designed using the CRISPR design tool (http://chopchop.cbu.uib.no/). MARCH8’s sgRNA-1 (5′ - CACCGGCTCATCCCAACCTCTTATC) and sgRNA-2 (5′- CACCGGTGCGAGAGAAGGAGGACAC) were synthesized and cloned into the pX458 gRNA plasmid (a gift from Dr. Feng Zhang, Addgene plasmid # 48138), as described (Ran et al., 2013). Single clonal knockout of HEK293T and Huh7.5.1 cells were obtained using the PX458 vector that expresses Cas9 and sgRNA against MARCH8. Green fluorescent protein (GFP) positive single cells were sorted at 24 hours post-transfection using a BD InFlux Cell Sorter into 96-well plates and screened for knockout via Sanger sequencing and western blot, as described (Ran et al., 2013). Sequences of mutant cell lines: 1) Huh7.5.1: WT: 5′- GAAGAAGACGACCAGATAAGAGGTTGGG – 3′; MARCH8^KO^#1: 5′- GAAGAA GACGACCAGA—–GGTTGGG – 3′. 2) Huh7.5.1: Wt: 5′- TCAGCTCCGGCTCCGGTGTCCTCCTTCT – 3′; MARCH8^KO^ #2: 5′- TCA GCTCCGGCTCC—-TCCTTCCCTTCT – 3′. 3) HEK293T cells: Wt: 5′- TCAGCTCCGGCTCCGGTGTCCTCCTTCT – 3′; MARCH8^KO^: 5′- TCAGCTCC————-TCCTTCT – 3′.

#### Viability assays

Cells were incubated for 2 hours at 37°C with 10% alamarBlue reagent (TREK Diagnostic Systems). Fluorescence was detected by Tecan luminometer (Tecan) according to the manufacturers’ protocols.

#### NS2 protein expression and purification

rNS2 (92–216 aa)–GST fusion was expressed in E.coli C41 (DE3, Lucigen) and purified, as described (Barouch-Bentov et al., 2016). Cultures were grown to OD600 = 0.6, followed by induction with 0.1mM IPTG for 18 hours at 16°C. Cells were pelleted, lysed by 3 passes via an EmulsiFlex-C5 (Avestin) in 50 mM HEPES, 300 mM NaCl, 1 mM DTT, 5 mM MgCl2, 0.5% Triton and protease inhibitors, and spun (48,000× g, 20 min). Lysates incubated with glutathione 4B-Sepharose beads at 4°C for 1 hour. After three washes, protein eluted in a buffer containing 20 mM glutathione. The GST tags cleaved by Thrombin (GE Healthcare).

#### *In vitro* ubiquitination assay

5 μg rNS2 was incubated with recombinant MARCH8, Huh7.5.1 cell extract, 2 μM ubiquitin–aldehyde, 0.5 μg/μl ubiquitin, 10 μM MG-132 and components of the ubiquitination kit (ENZO), including: E1 and E2 (UBE2H) enzymes and Mg-ATP in a final volume of 20 μL (Barouch-Bentov et al., 2016). After 2–6 hour incubation at 37°C, the reaction was stopped by addition of SDS sample buffer.

#### Detection of ubiquitination by IP

Cells co-transfected with GLuc-NS2 and/or GLuc-MARCH8 or HCV RNA and control cells were treated for 2 hours with 10 μM MG-132 and lysed in a buffer containing 100 mM Tris-HCl (pH 8.0), 0.15 M NaCl, 5 mM EDTA, 1% NP-40, 0.5% Triton X-100, DUB inhibitors (100 μM PR619, 5 mM 1, 10- phenanthroline, 5 mM NEM), and a protease inhibitor cocktail. Lysates were spun (14,000RPM, 10 min). ~300 μl reaction buffer (100 mM Tris-HCl (pH 8.0), 0.15 M NaCl, 5 mM EDTA) was added to ~300 μl lysis buffer. Anti-NS2 or IgG antibodies were then added to the clarified supernatants for ~2 hours followed by A/G Dynabeads and 16 hour incubation at 4°C, wash with Catch and release IP wash buffer (EMD Millipore) supplemented with 2M Urea, and elution in X5 SDS sample buffer.

#### Detection of ubiquitination using anti-K63 TUBE technology

Cells co-expressing GLuc-NS2 and GLuc-MARCH8 were treated with 10 μM MG-132 for 2 hours, washed with PBS, and lysed in the lysis buffer described above plus 500 nM FLAG® Anti-K63 TUBE reagent (LifeSensors). Clarified lysates were resuspended in reaction buffer composed of 500 nM FLAG® Anti-K63 TUBE reagent, 100 mM Tris-HCl (pH 8.0), 0.15 M NaCl, 5mM EDTA, 0.1% NP-40, 0.05% Triton X-100, and DUB inhibitors, and incubated for 1 hour on ice. IP with anti-NS2 antibody was conducted as described above.

#### NS2 stability assays

72 hours post-transfection of MARCH8^KO^ and WT Huh7.5.1 cells with HCV RNA J6/JFH, cells were treated with cycloheximide (100 μg/ml) for 8 hours and samples were collected every 2 hours. NS2 expression was analyzed in cell lysates via western blots and quantified by imageJ (NIH). The half-life of NS2 was calculated from the decay constant of the fitted exponential curves.

#### Core protein ELISA

The concentration of released core protein was measured in clarified cell culture supernatants by ELISA (Cell Biolabs) against standard curves of recombinant core antigen, according to the manufacturer’s instructions.

#### Density gradient centrifugations

Three days post-transfection with pFL-J6/JFH(p7-Rluc2A) RNA, clarified cell lysates were loaded on continuous 10%–60% sucrose gradients in TNE buffer and spun (16 hours, 230,500× g) at 4°C for isopycnic separation (Gastaminza et al., 2006). For rate zonal-like separation lysates were layered on top of 5%–35% sucrose density gradients in the presence or absence of 1% DDM and spun (1 hour, 270,000× g), as described (Gentzsch et al., 2013). The refraction index of 10–13 fractions collected was measured by a Milton-Roy refractometer. To detect core protein, ~200 μl of each fraction were incubated with 1% Triton for 30 minutes at 56°C. 1 μl of Heparin (1 mg/ml), 5.8 μl Nacl (5M) and 0.8 mL Methanol were then added, samples were vortexed, and proteins were precipitated by spinning for 5 min at 20,000G. Dried pellets were dissolved in 10 μl of 100 mM Tris buffer with 8M Urea and 40 μl 5X SDS sample buffer.

#### Proteolytic digestion protection assays

As described (Barouch-Bentov et al., 2016; Gentzsch et al., 2013), 72 hours following HCV transfection cells seeded in 6-well plates were scraped into 250 μL PK buffer (50 mM Tris-HCl pH 8.0; 10 mM CaCl2; 1 mM DTT) and subjected to freeze-thaw cycles. 50 μL samples were either left untreated or treated with 1% Triton X-100 for 5 minutes at RT and/or 30 or 50 μg/ml PK (Roche) for 1 hour on ice. PK digestion was terminated by addition of 5 mM PMSF and 10 minute incubation on ice. Level of residual core was determined by immunoblotting.

#### RNase digestion protection assays

As described (Barouch-Bentov et al., 2016; Gentzsch et al., 2013), 50 μL aliquots derived from gradient fraction #6 of the rate zonal centrifugation were either: 1. left untreated; 2. treated with S7 RNase (2U, 30 minutes at 37°C) (Roche); 3. pretreated with PK (50 μg/ml in 10X PK buffer for 1 hour on ice) followed by S7 RNase; or 4. pretreated with 1% Triton X-100 (5 minutes at RT) prior to PK and S7 RNase. PK activity was stopped by adding 10 mM PMSF and protease inhibitors prior to S7 RNase digestion. Total RNA was isolated from gradient fractions by NucleoSpin RNA virus kit (Macherey-Nagel). HCV RNA was quantified by qRT-PCR, as described (Neveu et al., 2012).

### QUANTIFICATION AND STATISTICAL ANALYSIS

The distributions of the absolute luminescence values measured in the primary screen were represented by boxplots. Whisker length corresponds to 1.5 times the interquartile range (IQR), which is equal to the difference between the third (Q3) and first (Q1) quartiles (IQR = Q3-Q1). Outliers above the upper whisker (Q3+1.5xIQR) were defined as positive interactions. To normalize the PCAs screen data we fit a Gaussian function to the distribution of RRS measurements. A Z-score was calculated for each interaction following a log-arithmic transformation by subtracting the mean value of this fit from the interaction signal and dividing the resulting value by the SD of the fit. *P values* were calculated by two-tailed unpaired t test or one- or two-way ANOVA with either Dunnett’s or Tukey’s post hoc tests.

### SOFTWARE AVAILABILITY

All software and programs used in this paper are available freely and are discussed in detail above. See the Key Resources Table for the relevant references.

## Supplementary Material

Supp2

Supp1

## Figures and Tables

**KEY RESOURCES TABLE:** 

REAGENT or RESOURCE	SOURCE	IDENTIFIER
Antibodies		
MARCH8 polyclonal antibody (A01)	Abnova	Cat#H00220972-A01; RRID:AB_463085
Anti-Hepatitis C Virus Core 1b antibody (C7–50)	Abcam	Cat#ab2740
Monoclonal anti-FLAG M2 antibody	Sigma-Aldrich	Cat#F3165; RRID:AB_259529
Mouse Ub antibody (P4D1)	Santa Cruz Biotechnology	Cat#SC-8017; RRID:AB_628423
Anti GLuc antibody	New England BioLabs	Cat#E8023
Monoclonal Anti-HA (HA-7) antibody	Santa Cruz Biotechnology	Cat#H3663
Normal mouse IgG	Santa Cruz Biotechnology	Cat#SC-2025; RRID:AB_737182
Normal Rabbit IgG Polyclonal Antibody control	Millipore, Sigma	Cat#12–370
Anti-mouse IgG, HRP-linked Antibody	Cell Signaling	Cat#7076S; RRID:AB_330924
Anti-Rabbit IgG HRP-linked	Cell Signaling	Cat#7074s
Goat anti-Rabbit IgG (H+L), Alexa Fluor 488	Invitrogen	Cat#A27034
Donkey anti-Mouse IgG (H+L) Alexa Fluor 568	Invitrogen	Cat#A10037; RRID:AB_2534013
Rabbit monoclonal anti-NS2	Gift from Dr. Brett Lindenbach	Barouch-Bentov et al., 2016
CellLight ER-GFP, BacMam 2.0	Invitrogen	Cat#C10590
Bacterial and virus strains		
pFL-J6/JFH(p7-Rluc2A), *Renilla* reporter HCV	Gift from Dr. Charles Rice	Murray et al., 2007
pDENV2 (New Guinea C strain), *Renilla* reporter	Gift from Dr. Pei-Yong Shi	Xie et al., 2013; Zou et al., 2011
pZIKV (FSS13025, Asian strain) *Renilla* reporter	Gift from Dr. Pei-Yong Shi	Shan et al., 2016
Chemicals, Peptides, and Recombinant Proteins		
PR619	LifeSensors	Cat#SI9619
1, 10- phenanthroline	LifeSensors	Cat#SI9649
Dithiobis-succinimidyl-propionate (DSP)	Pierce	Cat#22586
Protease Inhibitor Cocktail	Sigma-Aldrich	Cat#P8340
N-Ethylmaleimide (NEM)	Sigma-Aldrich	Cat#E3876
Lipofectamine 3000	Life Technologies	Cat#L3000–001
Cycloheximide (CHX)	Sigma-Aldrich	Cat#C7698
siIMPORTER	Millipore	Cat# 64–101
Dynabeads protein A/G	Life technology	Cat#10002d; Cat# 10004d
AlamarBlue	Invitrogen	Cat#DAL1100
Anti-K63 TUBEs	LifeSensors	Cat#UM604
Recombinant MARCH8	Oregene	Cat#TP305477
Recombinant NS2	Einav lab	Barouch-Bentov et al., 2016
Critical Commercial Assays		
Ubiquitinylation kit	ENZO	Cat#BML-UW9920–0001
Quickchange Lightning kit	Agilent	Cat# 210519
QuickTiter HCV Core Antigen ELISA Kit	Cell BioLabs	Cat#VPK-151
NucleoSpin RNA Virus kit	MACHEREY-NAGEL	Cat# 740956.250
Power SYBR Green RNA-to-CT 1-Step Kit	Applied Biosystems	Cat# 4389986
Experimental Models: Cell Lines		
Homo sapiens: HEK293T	ATCC	CRL-3216
Homo sapiens: Huh7.5	Apath, LLC	N/A
Homo sapiens: Huh7.5.1	Apath, LLC	N/A
Homo sapiens: HEK293T MARCH8KO	This paper	N/A
Homo sapiens: Huh7.5.1 MARCH8KO#1	This paper	N/A
Homo sapiens: Huh7.5.1 MARCH8KO#2	This paper	N/A
Experimental Models: Organisms/Strains		
E.coli C41 (DE3) Competent Cells	Lucigen	Cat#60442–1
E.coli DH5α Competent Cells	Thermofisher-Scientific	Cat#18265017
Oligonucleotides		
ON-TARGETplus SMART pools siRNA library, see Table S5	GE Healthcare Dharmacon	N/A
MARCH8 knockdown siRNA-1	Life Technologies	Cat# s47920
MARCH8 knockdown siRNA-2	Life Technologies	Cat# s47921
MARCH8 sgRNA-1	This paper	5′- CACCGGCTCATCCCAACC TCTTATC
MARCH8 sgRNA-2	This paper	5′- CACCGGTGCGAGAGAAGG AGGACAC
Primers for qPCR analysis, see Table S6	This paper	N/A
Recombinant DNA		
pMARCH8-LD mutant	This paper	van de Kooij et al., 2013
pGLuc-NS2 4KE mutant	Einav lab	Barouch-Bentov et al., 2016
pFLAG-MARCH8	This paper	N/A
pRAB7-GFP	Gift from Dr. Suzanne Pfeffer	N/A
pHA-UB	Gift from Dr. Brett Lindenbach	Barouch-Bentov et al., 2016
pHA-UBK48R mutant	Gift from Dr. Brett Lindenbach	N/A
pHA-UBK63R mutant	Gift from Dr. Brett Lindenbach	Barouch-Bentov et al., 2016
pX458 gRNA	Gift from Dr. Feng Zhang	Addgene plasmid # 48138
Unique ORFs in the UPS library, see Table S1	This paper	N/A
Software and Algorithms		
Prism 7	Graphpad	https://www.graphpad.com/scientificsoftware/prism/
CRISPR design tool	Chopchop	http://chopchop.cbu.uib.no/
Cytoscape	Cytoscape	https://cytoscape.org/
ImageJ	NIH	https://imagej.nih.gov/ij/

## Highlights

- Ubiquitin signaling factors interact with NS2 and play a role in HCV infection
- MARCH8 mediates K63-linked ubiquitination of HCV NS2 and ESCRT recruitment
- MARCH8 is required for *Flaviviridae* infection and intracellular envelopment of HCV
- MARCH8 is a potential target for host-targeted antiviral strategies
