# Supplementary material for: MARCH8 Ubiquitinates the Hepatitis C Virus Nonstructural 2 Protein and Mediates Viral Envelopment
Source: Cell Rep. Author manuscript; Available in PMC 2020 Mar 3. (PMC7053169; doi:10.1016/j.celrep.2019.01.075)
Supplement: Supp1 [file NIHMS1013108-supplement-Supp1.pdf]

Cell Reports, Volume *26*

Supplemental Information

MARCH8 Ubiquitinates the Hepatitis C Virus Nonstructural 2 Protein and Mediates Viral Envelopment

Sathish Kumar, Rina Barouch-Bentov, Fei Xiao, Stanford Schor, Szuyuan Pu, Elise Biquand, Albert Lu, Brett D. Lindenbach, Yves Jacob, Caroline Demeret, and Shirit Einav

Supplemental Information

Figure S1

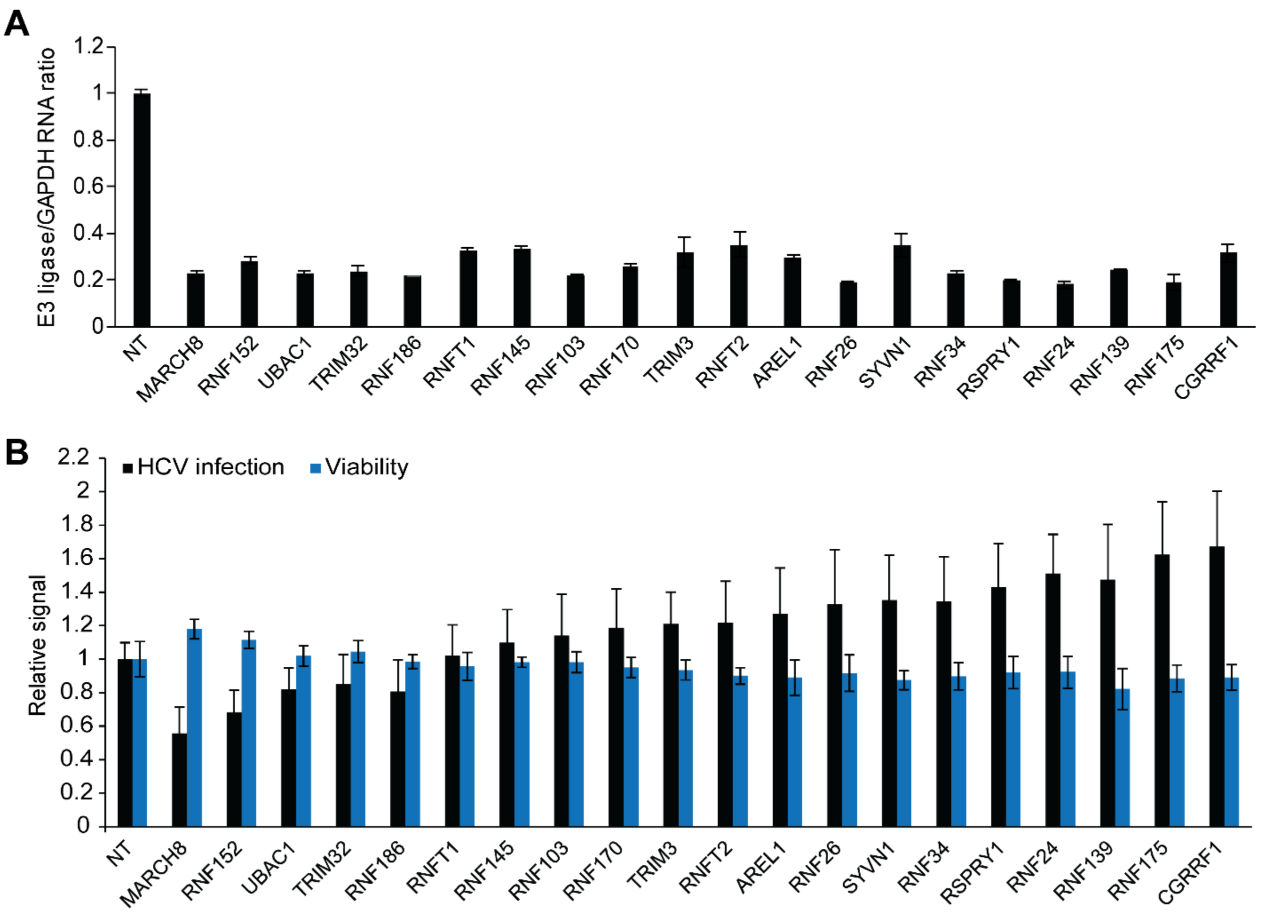

Figure S1. Testing the involvement of E3 ligases interacting with NS2 in HCV infection via an siRNA screen, Related to Figure 2.

A. E3 ligase/GAPDH RNA ratio relative to NT control measured by qRT-PCR at 72 hr post siRNA transfection.

B. Overall HCV infection (black) measured via luciferase assays and cell viability (blue) measured via alamarBlue assays at 72 hr post-infection of siRNA-transfected Huh7.5.1 cells. Data are expressed relative to NT siRNA. Data are an average of two independent screens with 8 replicates each.

Figure S2

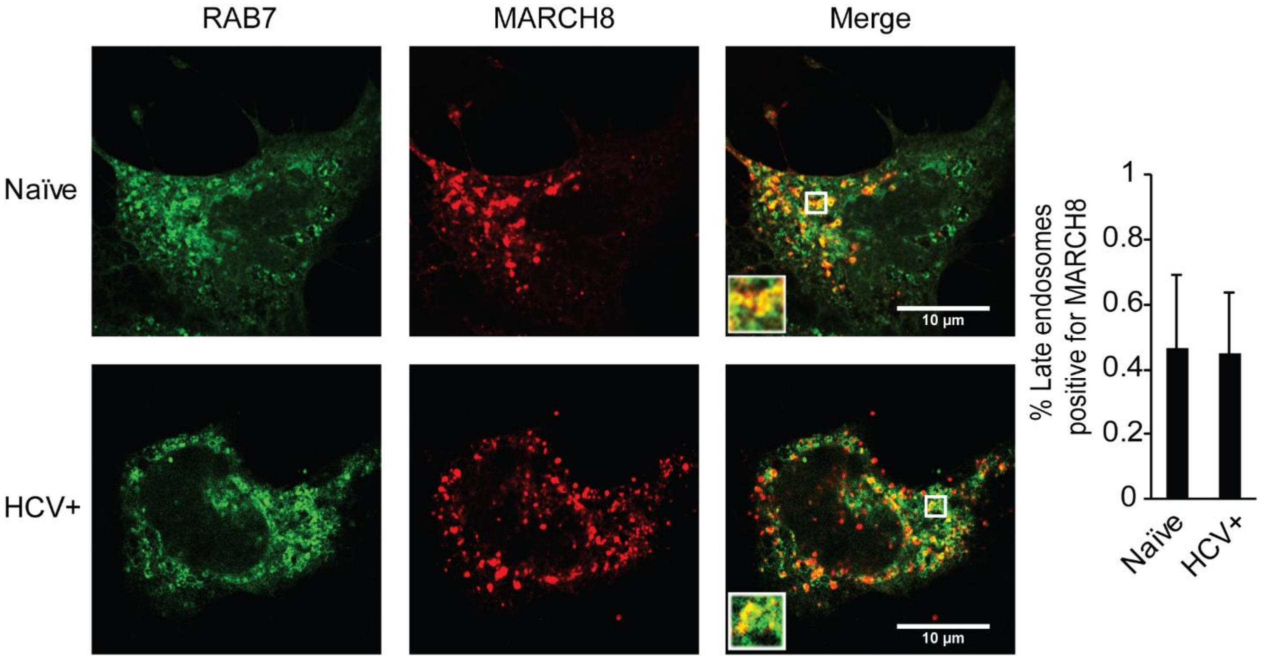

Figure S2. MARCH8 localization to late endosomes is not altered in HCV RNA-transfected cells, Related to Figure 3.
Confocal IF microscopy images of RAB7 (green) and MARCH8 (red) in naïve and HCV (J6/JFH) RNA-transfected Huh7.5.1 cells ectopically expressing FLAG-MARCH8 48 hr post-transfection. Shown are representative images at 60X magnification and quantitative data (mean+/-s.d.). n=20 cells per category.

Figure S3

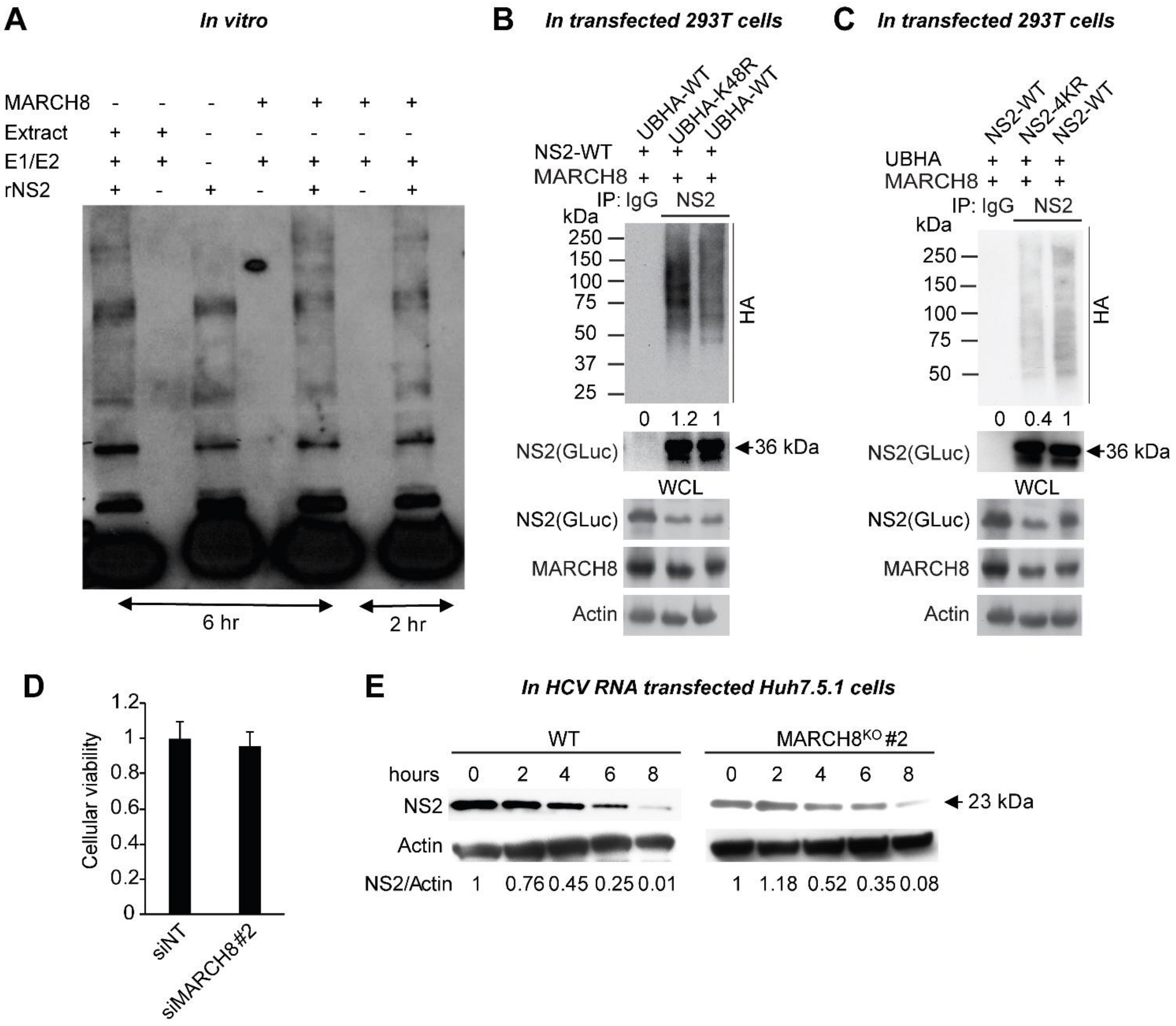

Figure S3. MARCH8 ubiquitinates NS2 without altering its stability, Related to Figure 4.
A. rNS2 was incubated with ubiquitin for 2 or 6 hr in the presence or absence of E1 activating enzyme, UBE2H (E2 conjugating enzyme) and recombinant MARCH8 or Huh7.5.1 cell extract. MARCH8 and cell extracts incubated in the absence of NS2 served as controls. A representative membrane blotted with anti-NS2 antibody is shown.
B-C. Lysates of 293T cells co-transfected with WT GLuc-NS2 (B, C) or 4KR GLuc-NS2 mutant (C), UBHA-WT (B, C) or UBHA-K48R mutant (B), and MARCH8 plasmids were subjected to IP with anti-NS2 or IgG antibodies. Representative membranes blotted with antibodies against HA, GLuc, MARCH8 and actin and quantitative NS2 ubiquitination data normalized to NS2 pull down signal relative to the respective right lane are shown. WCL, whole cell lysates.
D. Cellular viability measured via alamarBlue assays in Huh7.5.1 cells 72 hr post-transfection with the indicated siRNA.
E. NS2 expression by Western blot at different time points following cycloheximide treatment in WT and MARCH8^{KO} Huh7.5.1 cells transfected with HCV RNA cells (treatment was initiated at 72 hr post-transfection). The experiment was conducted twice. Representative membranes blotted with anti-NS2 and anti-actin antibodies and NS2/actin protein ratio are shown.

Figure S4

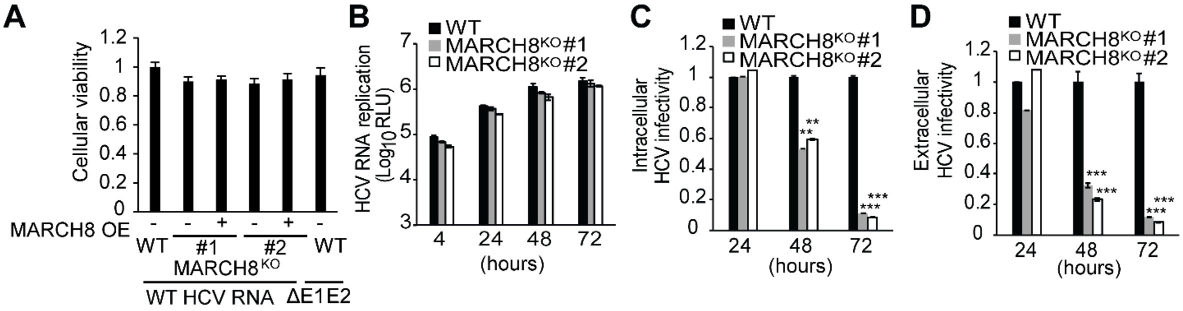

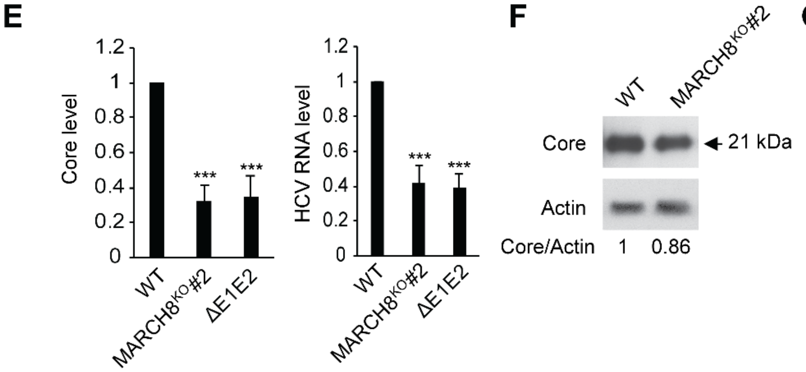

G

Samples	Specific infectivity (PFU/RNA copies)
WT	1.77±1.3 x10^{-5}
MARCH8^{KO}#2	1.19±3.0 x10^{-8}
ΔE1E2	0

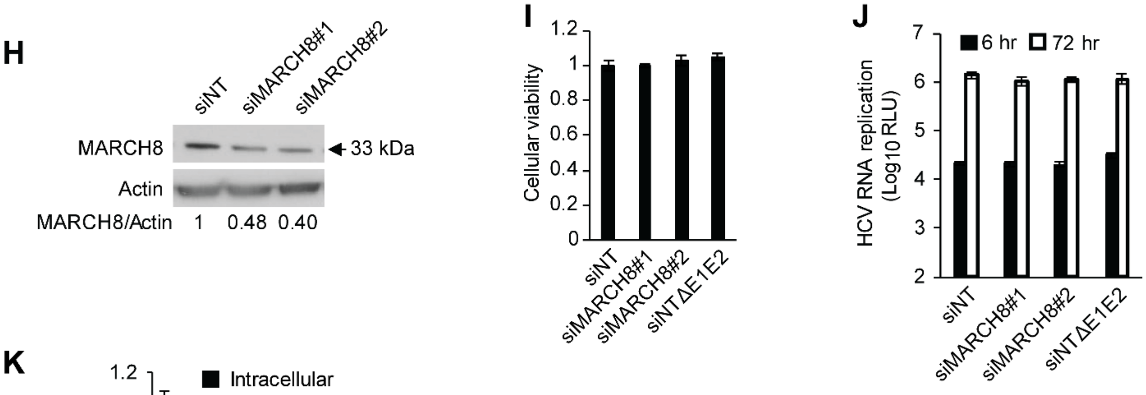

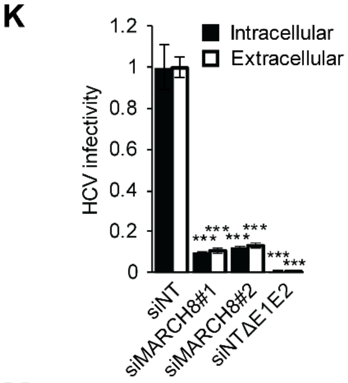

L

	TCID$_{50}$/ml	
	Intracellular	Extracellular
siNT	2.69x10^5	3.95x10^5
siMARCH8#1	3.95x10^3	4.56x10^3
siMARCH8#2	7.49x10^2	1.88x10^3
ΔE1E2	0	0

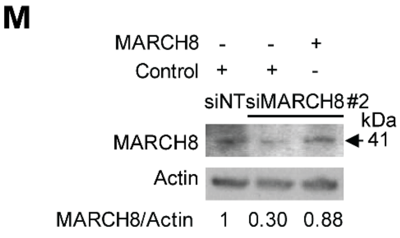

N

	OE	TCID$_{50}$/ml	
		Intracellular	Extracellular
siNT	Control	1.25x10^5	7.29x10^4
siMARCH8#2	Control	2.69x10^3	1.25x10^2
siMARCH8#2	MARCH8	2.34x10^5	1.83x10^5

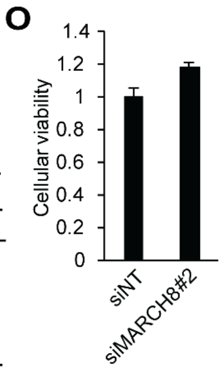

Figure S4. MARCH8 mediates HCV assembly, Related to Figure 5.
A. Cellular viability measured via alamarBlue assays in the indicated Huh7.5.1 cell lines 72 hr post-transfection with WT HCV RNA or an assembly defective, E1 and E2 glycoprotein deleted HCV mutant (ΔE1-E2). OE, over-expression.
B. HCV RNA replication in the indicated cell lines 4, 24, 48 and 72 hr post-electroporation with WT HCV RNA measured via luciferase assays (RLU, relative light units).
C-D. Intra- (C) and extracellular (D) infectivity measured via luciferase assays in naïve cells inoculated with clarified cell lysates or supernatants derived from the HCV electroporated cell lines, respectively, at 24, 48 and 72 hr post-transfection.
E. HCV core protein (left) and viral RNA (right) released into the culture supernatant at 72 hr post-electroporation measured by ELISA and qRT-PCR, respectively.
F. Intracellular core protein level and core to actin protein ratio in the indicated cell lines by Western blot.
G. Specific infectivity (focus-forming unit/viral RNA molecules) in the indicated cell lines.
H. MARCH8 levels and MARCH8/actin protein ratio in Huh7.5.1 cells at 72 hr post-transfection with the indicated siRNAs by Western blot.
I. Cellular viability measured via alamarBlue assays in Huh7.5.1 cells 72 hr post-transfection with the indicated siRNAs.
J. HCV RNA replication in MARCH8 depleted Huh7.5.1 cells 6 and 72 hr post-electroporation with WT HCV RNA or the ΔE1-E2 mutant measured by luciferase assays.
K. HCV infectivity measured via luciferase assays by inoculating naïve cells with lysates (intracellular) and supernatants (extracellular) harvested at 72 hr post-electroporation.
L. Intra- and extracellular viral titers measured by limiting dilution assays. $TCID_{50}$, 50% tissue culture infectious dose.
M-N. Levels of MACRH8 by Western blot analysis at 72 hr post siRNA transfection (M) and intra- and extracellular viral titers measured by limiting dilution assays (N) in cells co-transfected with MARCH8 siRNA and a plasmid encoding siRNA-resistant MARCH8 or control.
O. Cellular viability measured via alamarBlue assays in Huh7 cells 72 hr post-transfection with the indicated siRNAs.
J and K represent data pooled from three independent experiments with 3-6 biological replicates each. Remaining panels are representative experiments out of two conducted. Shown are mean +/- s.d.; **p<0.01, ***p<0.001 relative to corresponding WT or NT controls (One-way (A, E, I, K) or two-way (B, C, D, J) ANOVA with Dunnett's post-hoc tests, or Student's t-test (O)).

Figure S5

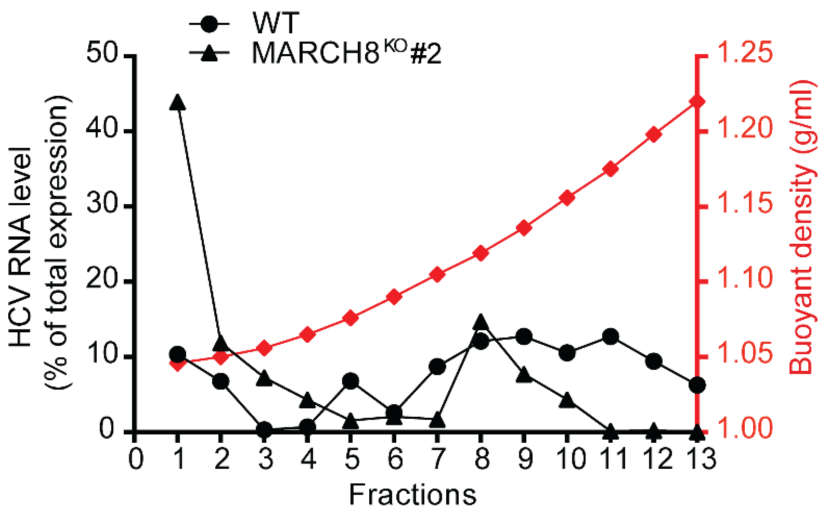

Figure S5. MARCH8 is required for HCV envelopment, Related to Figure 7.
Sucrose density gradient analysis of intracellular viral particles derived from control (WT) and MARCH8^{KO} cells 3 days post-transfection with HCV RNA by isopycnic separation. Thirteen fractions were collected and analyzed. The experiment was conducted twice. Plotted are the buoyant density along the gradient (right axis) and the percentage of HCV RNA normalized to the total RNA level (left axis).

Table S1. Unique ORFs included in the UPS library arranged by categories, Related to Figure 1.
Please see a separate Excel file.

Table S2. Random Reference Set (RRS1) tested by HT-GPCAs in the primary screen, Related to Figure 1.

Gene Symbol (Interactor B)	Entrez Gene ID (Interactor B)	Average Z-Score
ACTC1	70	1.27
CORO1B	57175	1.80
VCL	7414	1.45
KRAS	3845	0.86
PFN4	375189	0.34
RHOC	389	1.10
ACTR3	10096	1.40
WASL	8976	1.31
RDX	5962	1.40
DIAPH3	81624	0.44
ARPC1A	10552	0.96

Table S3. Known positive interactions of NS2 tested by PCAs, Related to Figure 1.

Gene Symbol (Interactor B)	Entrez Gene ID (Interactor B)	Average Z-Score	Reference
CIDEB	27141	3.17	(Cai et al., 2016)
CTSB	1508	2.80	(Einav et al., unpublished)
HRS	9146	4.45	(Barouch-Bentov et al., 2016)
AP1M1	8907	2.86	(Xiao F, 2018)
AP4M1	9179	2.34	(Xiao F, 2018)

Table S4. UPS-NS2 interactions by PCAs (secondary screen), Related to Figure 1.
The average z-scores measured by PCAs in a representative experiment.

S.No	Protein	Entrez Gene ID	Average Z-score	Function
1	ANAPC5	51433	-0.13	APC/CDC complex
2	ASB11	140456	16.95	E3 CRL adaptor
3	ASB14	142686	9.55	E3 CRL adaptor
4	ASB5	140458	2.27	E3 CRL adaptor
5	ASB6	140459	0.66	E3 CRL adaptor
6	ATXN3	4287	0.85	DUB
7	BIRC8	112401	0.97	E3-RING
8	BTRC	8945	5.32	E3 CRL adaptor
9	CGRRF1	10668	9.17	E3-RING
10	CYHR1	50626	6.98	E3-RING
11	DCAF15	90379	0.61	E3 CRL adaptor
12	DDB1	1642	0.40	E3 CRL adaptor
13	FBXL16	146330	0.24	E3 CRL adaptor
14	FBXL4	26235	3.43	E3 CRL adaptor
15	FBXO21	23014	0.26	E3 CRL adaptor
16	FBXO4	26272	1.46	E3 CRL adaptor
17	FBXO7	25793	3.41	E3 CRL adaptor
18	FBXW11	23291	3.43	E3 CRL adaptor
19	FBXW12	285231	0.92	E3 CRL adaptor
20	AREL1	9870	37.30	E3-HECT
21	KLHL11	55175	1.13	E3 CRL adaptor
22	KLHL21	9903	34.37	E3 CRL adaptor
23	KLHL26	55295	1.35	E3 CRL adaptor
24	KLHL29	114818	0.99	E3 CRL adaptor
25	KLHL34	257240	0.76	E3 CRL adaptor
26	MARCH2	51257	0.63	E3-RING
27	MARCH3	115123	0.70	E3-RING
28	MARCH8	220972	2.22	E3-RING
29	NDFIP1	80762	4.90	E3-HECT regulator
30	NDFIP2	54602	12.15	E3-HECT regulator
31	OTUD6A	139562	0.79	DUB
32	PAN2	9924	0.14	DUB
33	PCBP2	5094	14.07	E3-regulator
34	PSMD14	10213	0.43	DUB
35	RBX1	9978	3.59	E3 CRL component
36	RNF103	7844	3.89	E3-RING
37	RNF139	11236	6.62	E3-RING
38	RNF145	153830	4.98	E3-RING

39	RNF152	220441	2.60	E3-RING
40	RNF170	81790	4.07	E3-RING
41	RNF175	285533	8.58	E3-RING
42	RNF185	91445	1.26	E3-RING
43	RNF186	54546	2.57	E3-RING
44	RNF208	727800	1.64	E3-RING
45	RNF24	11237	12.35	E3-RING
46	RNF26	79102	21.17	E3-RING
47	RNF34	80196	6.40	E3-RING
48	RNF5	6048	1.29	E3-RING
49	RNFT1	51136	2.24	E3-RING
50	RNFT2	84900	3.33	E3-RING
51	RSPRY1	89970	5.76	E3-RING
52	SYVN1	84447	19.91	E3-RING
53	TAX1BP1	8887	0.53	Other
54	TRAF3IP2	10758	-0.36	Other
55	TRAF6	7189	0.07	E3-RING
56	TRIM3	10612	2.48	E3-RING
57	TRIM32	22954	2.76	E3-RING
58	UBAC1	10422	3.06	E3-RING
59	UBE2J1	51465	4.46	E2
60	UEVLD	55293	0.74	E2
61	USP10	9100	2.73	DUB
62	USP11	8237	0.83	DUB
63	USP19	10869	0.74	DUB
64	USP21	27005	1.09	DUB
65	USP25	29761	0.27	DUB
66	USP28	57646	-0.05	DUB
67	USP30	84749	2.24	DUB
68	USP38	84640	6.40	DUB
69	USP5	8078	0.44	DUB
70	USP8	9101	7.09	DUB
71	WDR82	80335	0.93	E3 CRL adaptor
72	ZBTB32	27033	0.33	E3 CRL adaptor

Table S5. siRNA library targeting the 20 E3 ligases that interact with NS2, Related to Figure 2.
NA is non-applicable.

Pool Catalog Number	Duplex Catalog Number	Gene Symbol	GENE ID	Gene Accession	GI Number	Sequence
D-001810-10	D-001810-01	Non-targeting	NA	NA	NA	UGGUUUACAUGUCGACUAA
D-001810-10	D-001810-02	Non-targeting	NA	NA	NA	UGGUUUACAUGUUGUGUGA
D-001810-10	D-001810-03	Non-targeting	NA	NA	NA	UGGUUUACAUGUUUUCUGA
D-001810-10	D-001810-04	Non-targeting	NA	NA	NA	UGGUUUACAUGUUUUCCUA
L-007184-00	J-007184-06	AREL1	9870	NM_014821	42734314	GCGCAAGGCUGGGCGUUAU
L-007184-00	J-007184-07	AREL1	9870	NM_014821	42734314	CGAAGAAGGUGUACUGCUA
L-007184-00	J-007184-08	AREL1	9870	NM_014821	42734314	GCAUUUACUUUGAGGCUUA
L-007184-00	J-007184-09	AREL1	9870	NM_014821	42734314	GGGAAUGGUUUGAGCUAAU
L-007060-00	J-007060-05	RNF26	79102	NM_032015	34878803	GCCGAGAGAGGCUCAAUGA
L-007060-00	J-007060-06	RNF26	79102	NM_032015	34878803	CGUAGUGGCUGCCUUCCUA
L-007060-00	J-007060-07	RNF26	79102	NM_032015	34878803	GCACUGAAAUCCUGAUGCG
L-007060-00	J-007060-08	RNF26	79102	NM_032015	34878803	UGUGUACUCUGCUGUAUAG
L-007090-00	J-007090-05	SYVN1	84447	NM_032431	51317310	UCAUCAAGGUUCUGCUGUA
L-007090-00	J-007090-06	SYVN1	84447	NM_032431	51317310	GAGAAGAGAUGGUGACUGG
L-007090-00	J-007090-07	SYVN1	84447	NM_032431	51317310	CAACAUGAACACCCUGUAU
L-007090-00	J-007090-08	SYVN1	84447	NM_032431	51317310	GGAAAGGCCUCCAGCUCCU
L-006943-00	J-006943-05	RNF24	11237	NM_007219	10518498	GCUCGGAUUUCCCACAUUA
L-006943-00	J-006943-06	RNF24	11237	NM_007219	10518498	GGGCAGAGAACAUUGUAUA
L-006943-00	J-006943-07	RNF24	11237	NM_007219	10518498	ACAGAAAGUGCCUUAUUAA
L-006943-00	J-006943-08	RNF24	11237	NM_007219	10518498	GAAUUUACAUGAGCUCUGU
L-006933-00	J-006933-05	CGRRF1	10668	NM_006568	50726998	ACAGAUUGCCUUGAAGAUA
L-006933-00	J-006933-06	CGRRF1	10668	NM_006568	50726998	GUACCCAGAUCUCGCUAUC
L-006933-00	J-006933-07	CGRRF1	10668	NM_006568	50726998	GAGGAUGACCGGGAAAUUU
L-006933-00	J-006933-08	CGRRF1	10668	NM_006568	50726998	GUAUUGGGAUGGUUUGGUU
L-007170-00	J-007170-05	RNF175	285533	NM_173662	27734858	CAUUUGGUGUUGUGGGUUA
L-007170-00	J-007170-06	RNF175	285533	NM_173662	27734858	GGGAUGUUCUCCGUUAUUA
L-007170-00	J-007170-07	RNF175	285533	NM_173662	27734858	GACAAUAUCUGUGCAGUCU
L-007170-00	J-007170-08	RNF175	285533	NM_173662	27734858	CACGAUUGGUCUACAAAUG
L-006942-00	J-006942-05	RNF139	11236	NM_007218	38045935	GGGAAAAGCUUGACGAUUA
L-006942-00	J-006942-06	RNF139	11236	NM_007218	38045935	AGAGAGACUUUACUGUUUA
L-006942-00	J-006942-07	RNF139	11236	NM_007218	38045935	GGGAGCCGCUUACAAGAAA
L-006942-00	J-006942-08	RNF139	11236	NM_007218	38045935	UGACAGGCGUCUUGGCUUU
L-022564-01	J-022564-09	RSPRY1	89970	NM_133368	45387948	CAAGUCAGUAUCCGAGAAA
L-022564-01	J-022564-10	RSPRY1	89970	NM_133368	45387948	CUGAUUAUCUGAAACGUCA
L-022564-01	J-022564-11	RSPRY1	89970	NM_133368	45387948	CAGUAUUAGUGACCGGCUU
L-022564-01	J-022564-12	RSPRY1	89970	NM_133368	45387948	GCAAUGAUGUCAGCGAGUA
L-007072-00	J-007072-05	RNF34	80196	NM_025126	37595536	UCUGAGAAAUAUACCCAUA
L-007072-00	J-007072-06	RNF34	80196	NM_025126	37595536	CGGCACAGGUACAAAGUGA
L-007072-00	J-007072-07	RNF34	80196	NM_025126	37595536	GGCCCAACAUAGUUUGUAA
L-007072-00	J-007072-08	RNF34	80196	NM_025126	37595536	GCUUAUGGAUGGAGACCAA
L-007146-00	J-007146-05	RNF145	153830	NM_144726	21389514	UGACAAGUAUUGCGGAAUG
L-007146-00	J-007146-06	RNF145	153830	NM_144726	21389514	CAGAAUUGCUAGUAGUUUA
L-007146-00	J-007146-07	RNF145	153830	NM_144726	21389514	UGGUUCAGCUUUAUCUAUA
L-007146-00	J-007146-08	RNF145	153830	NM_144726	21389514	GAGAAUUGGUUCAGGUAGU
L-006594-00	J-006594-05	RNF103	7844	NM_005667	37595534	UUACCAAUGUGGCGAUUUA
L-006594-00	J-006594-06	RNF103	7844	NM_005667	37595534	GUUCAUGUGCCAAUAAAUA
L-006594-00	J-006594-07	RNF103	7844	NM_005667	37595534	GAGCUUGGUUCUAGUUAAU
L-006594-00	J-006594-08	RNF103	7844	NM_005667	37595534	GCCAUUGUGUGGUAUGAAA
L-007078-00	J-007078-05	RNF170	81790	NM_030954	21361953	GGGCAACCCAGAUCUAUUA
L-007078-00	J-007078-06	RNF170	81790	NM_030954	21361953	GGCCAAAUAUCAAGGUGAA
L-007078-00	J-007078-07	RNF170	81790	NM_030954	21361953	UAGACAAACGGUAACCUUA
L-007078-00	J-007078-08	RNF170	81790	NM_030954	21361953	GAUCUACCCACUUUACUGA
L-007097-01	J-007097-09	RNFT2	84900	NM_032814	14249505	GGACGUGUGGGCGGAGUUA

L-007097-01	J-007097-10	RNFT2	84900	NM_032814	14249505	UGAAGGGGCACAAGAAAUU
L-007097-01	J-007097-11	RNFT2	84900	NM_032814	14249505	GACGGAACCCAGUGUAUUA
L-007097-01	J-007097-12	RNFT2	84900	NM_032814	14249505	GUAGGUUACUAGUGAAUAC
L-006931-00	J-006931-05	TRIM3	10612	NM_033278	32454736	GUACAGCACAGGCGGCAAA
L-006931-00	J-006931-06	TRIM3	10612	NM_033278	32454736	GCACAUAUGAGCUAGUGUA
L-006931-00	J-006931-07	TRIM3	10612	NM_033278	32454736	GAGCGCCACUGCACACGAA
L-006931-00	J-006931-08	TRIM3	10612	NM_033278	32454736	GAAUGAAAUUGUAGUAACG
L-010205-00	J-010205-05	RNFT1	51136	NM_016125	21361528	GAUAAACUCCAUACCUAAA
L-010205-00	J-010205-06	RNFT1	51136	NM_016125	21361528	AAUCUAAGGGUUACUGGUA
L-010205-00	J-010205-07	RNFT1	51136	NM_016125	21361528	GCGUCAAACUUGUUAUGCA
L-010205-00	J-010205-08	RNFT1	51136	NM_016125	21361528	AAUGCAAGCUCCAGAAAUA
L-006950-01	J-006950-09	TRIM32	22954	NM_012210	15208649	CCAAAUAGGACACACGAUG
L-006950-01	J-006950-10	TRIM32	22954	NM_012210	15208649	GUGAAGUACUAGUCGCUGA
L-006950-01	J-006950-11	TRIM32	22954	NM_012210	15208649	GGACAGUUAACGUGGAAGA
L-006950-01	J-006950-12	TRIM32	22954	NM_012210	15208649	UGGUAACUAUCGUAUACAA
L-020776-01	J-020776-09	UBAC1	10422	NM_016172	55770883	GCUAAUUGAACACGCAGAA
L-020776-01	J-020776-10	UBAC1	10422	NM_016172	55770883	GCACGUAGGUGGCGUUGUU
L-020776-01	J-020776-11	UBAC1	10422	NM_016172	55770883	CAGAAUGCCGCGUGCGAGU
L-020776-01	J-020776-12	UBAC1	10422	NM_016172	55770883	AGAGAUGAGCUGACGGAAA
L-007161-00	J-007161-06	MARCH8	220972	NM_001002	50539413	GAAUGGCCCUUUUGGACUA
L-007161-00	J-007161-07	MARCH8	220972	NM_001002	50539413	GAGCAGAAAUCAUUCACGU
L-007161-00	J-007161-08	MARCH8	220972	NM_001002	50539413	GGAAGAGACUCAAGGCCUA
L-007161-00	J-007161-09	MARCH8	220972	NM_001002	50539413	UAAAGUGUAUGUGCAAUUG
L-007160-02	J-007160-05	RNF152	220441	NM_173557	91754187	CACAACAUGUCUUGCAUUU
L-007160-02	J-007160-06	RNF152	220441	NM_173557	91754187	GGUGUGCACUGUCAUCUUG
L-007160-02	J-007160-07	RNF152	220441	NM_173557	91754187	GAUAUCCUGUGGCUGAAGA
L-007160-02	J-007160-17	RNF152	220441	NM_173557	91754187	GUGCGGUCUCAACAAGAUA
L-006999-00	J-006999-05	RNF186	54546	NM_019062	9506662	GCACCAAGACCCUGCAACA
L-006999-00	J-006999-06	RNF186	54546	NM_019062	9506662	GACAACACCUGGUCCAUCA
L-006999-00	J-006999-07	RNF186	54546	NM_019062	9506662	CAUCUACCCGGGUGUCUUA
L-006999-00	J-006999-08	RNF186	54546	NM_019062	9506662	AGCCACAUCUCUUCCAUUG

Table S6. **Taqman reagents used in this paper, Related to Figure 2.**

S.No	Gene	Forward primer	Reverse primer
1	MARCH8	AGTGACATTCCACGTCATTGC	GATCTCCTCAGCAGTACGGTC
2	RNF152	CTGTCATCGCCATTCCACACA	GCAGCATGTAGCACCCATTG
3	UBAC1	AGAAGGCGAATGCAATGCTG	CGTGTTCAATTAGCCACTCCA
4	TRIM32	CCGGGAAGTGCTAGAATGCC	CAGCGGACACCATTGATGCT
5	RNF186	CCAGCCATGCACAGAGGTATC	CGTGGTTTGCACTTACTTCATCC
6	RNFT1	CCTGAAGCAAAGACATCTGGG	ACTGTGCAGTTGGCTACGATT
7	RNF145	AGTGAACTGGAGTTTGCCTATG	ACACACCACCAACTGACCTATT
8	RNF103	CATTGTGTGGTATGAAACTGGCA	CCCGGCACTCCAAAATGGT
9	RNF170	TGGAGACCAACTGTGGACATC	CCCCAAGCCATGAACCATATC
10	TRIM3	GCGACCTGGAGACCATTTGT	GCTACTGCCGATGTGTTCCTG
11	RNFT2	CACAGCAGCAACACGGATAAC	CTGCCTGATAAGCCCGAGA
12	AREL1	TCTGTGGTTGCATTCTTCTTCAC	TCGCGGTCCTCATTCTGGA
13	RNF26	GGGTATTCTCAGTTAGGACCCG	TCCTCCTCATTGAGCCTCTCT
14	SYVN1	CTTCACCGTTTTTCGGGATGA	CCAGGAGGAACATAAGAGAGACA
15	RNF34	GGAGAGCTTATGGATGGAGACC	GGTTCCGATCCTCTGCGTT
16	RSPRY1	CCCACTGCTGACACAAGGAG	ACACTAGCCCATCCACATTTTG
17	RNF24	GGATTTGCCCATGTAAGCACG	CAGGGGACACACTTTACGAAC
18	RNF139	ACAACACGTCAGCTTTTGGAA	CTGTAGAACGATAAGTGCCATCC
19	RNF175	AGCTACCCGAAAACCCCTCT	TCGCCAAGTAACCCACAACAC
20	CGRRF1	TGAAGATAGCCTCCTTACATGCT	TCTAATGCTTGGGGAGTGCTTAT
